# TGF-β1 promotes colorectal cancer immune escape by elevating B7-H3 and B7-H4 *via* the miR-155/miR-143 axis

**DOI:** 10.18632/oncotarget.11950

**Published:** 2016-09-10

**Authors:** Xinru Zhou, Yong Mao, Jianjie Zhu, Fanyi Meng, Qi Chen, Lihua Tao, Rui Li, Fengqing Fu, Cuiping Liu, Yuanjia Hu, Weipeng Wang, Hongjian Zhang, Dong Hua, Weichang Chen, Xueguang Zhang

**Affiliations:** ^1^ Center for Drug Metabolism and Pharmacokinetics, College of Pharmaceutical Sciences, Soochow University, Suzhou, China; ^2^ Department of Oncology, The Fourth Affiliated Hospital of Soochow University, Wuxi, China; ^3^ Department of Gastroenterology, The First Affiliated Hospital of Soochow University, Suzhou, China; ^4^ Jiangsu Institute of Clinical Immunology, The First Affiliated Hospital of Soochow University, Suzhou, China; ^5^ Institute of Chinese Medical Sciences, State Key Laboratory of Quality Research in Chinese Medicine, University of Macau, China

**Keywords:** colorectal cancer, tumor evasion, TGF-β1, co-inhibitor, microRNA

## Abstract

Transforming growth factor-beta 1 (TGF-β1) suppresses T cell function, promoting tumor immune escape. Yet, whether the depression of TGF-β1 on T cell function is mediated by co-inhibitory molecules B7-H3 and B7-H4 remains largely unclear. Here, we demonstrated that TGF-β1 elevated the expression of miR-155 in colorectal cancer cells through SMAD3 and SMAD4. The upregulated miR-155 attenuated miR-143 by inhibiting its direct target, the transcription factor CEBPB. Consequently, the direct target genes of miR-143, B7-H3 and B7-H4, were augmented in the cytoplasm and membrane of tumor cells. Over-expression of B7-H3 and B7-H4 in HCT-116 cells induced T cells to secrete TGF-β1 and the immunosuppressive cytokines IL-2, IL-6, and IL-17. Restoration of miR-143 inhibited the growth of HCT-116 xenograft tumors in mice, and also repressed the expression of B7-H3 and B7-H4 in the tumors. Thus, this study reveals the mechanism by which TGF-β1 leads to T cell-mediated tumor evasion through an increase in B7-H3 and B7-H4 expression.

## INTRODUCTION

Colorectal cancer remains one of the most common malignancies and the leading cause of death by cancer in the world [1]. Although great progress has been made in colorectal cancer therapy, improvements in conventional treatment modalities have had only a modest impact on survival. Thus, alternative strategies such as immunotherapy are now being considered. There is compelling evidence to show that cancer cells escape the host's immunity by actively developing multiple suppressive mechanisms within the cancer microenvironment [2]. For instance, tumor cells evade T cell surveillance by creating an immunosuppressive environment via the production of factors such as transforming growth factor β1 (TGF-β1) [3]. TGF-β1 levels are apparently boosted in colon, esophageal, gastric, hepatocellular and pancreatic cancer; and correlate with tumor progression, metastasis and angiogenesis, which result in poor prognostic outcome [4]. TGF-β1 is broadly immunosuppressive, since cytotoxic T lymphocytes exposed to TGF-β1 are unable to kill tumor cells in humans or mice [5]. TGF-β1 also promotes the generation of immunosuppressive regulatory T (Treg) cells [6] and increases the capacity of macrophages to produce the immunosuppressive cytokine IL-10 [7]. Additionally, TGF-β1 inhibits IFN-γ production in NK cells through Smad2, Smad3, and Smad4 and at least in part through inhibition of T-bet [8]. Elevated serum TGF-β1 levels are observed in metastatic stages of many cancers and correlate with poor prognoses [9]. However, the molecular mechanisms behind the immunosuppressive function of TGF-β1 are not fully elucidated. Specifically, the impact of TGF-β1 on cancer cells is still completely unknown.

The abnormal expression of B7/CD28 superfamily molecules in the cancer microenvironment has been determined as an important immunosuppressive mechanism in many types of human cancers [2]. Recently, four CD28 family receptors containing the co-stimulatory CD28 and ICOS, the co-inhibitory CTLA4 and PD-1 (PDCD1), as well as seven B7 family ligands including co-stimulatory CD80 (B7-1), CD86 (B7-2), and B7-H2 (ICOSLG, CD275), and co-inhibitory B7-H1 (PD-L1, CD274), B7-DC (PD-L2, CD273), B7-H3 (CD276), and B7-H4 (VTCN1, B7X), have been found to be expressed in various lymphoid or non-lymphoid tissues [10]. However, high levels of co-inhibitory molecules and limited co-stimulatory molecules are detected in the tumor microenvironment [2, 11, 12]. It is reasoned that this imbalance in the B7/CD28 pathway facilitates tumor immune evasion, yet whether and how these molecules are regulated by TGF-β1 to maintain immunosuppressive function are still unknown.

Recent studies have reported that microRNAs (miRNAs) are activated by TGF-β1 and play important roles in inducing epithelial-mesenchymal transition (EMT) and metastasis in cancers [13–16]. miRNAs are endogenous non-coding RNA of ~23 nucleotides, which inhibit protein translation by interacting with the 3′-untranslated regions (3′-UTRs) of the messenger RNAs (mRNAs) of target genes [17]. Although miRNAs have been increasingly described to be involved in the inhibition of B7/CD28 molecules [18–24], further investigations are needed to fully understand whether they participate in TGF-β1-mediated regulation of B7/CD28 molecules in colorectal cancer.

Our present study shows that TGF-β1 upregulates co-inhibitory molecules B7-H3 and B7-H4 in the membrane and cytoplasm of colorectal cells via the miR-155/mir-143 axis, which in turn induces T cells to secrete immunosuppressive cytokines to maintain a tumor microenvironment. Restoring the level of miR-143 and thus blocking this regulatory pathway suppresses tumor growth *in vitro* and *in vivo*. Our results identify a novel mechanism by which cancer cells crosstalk with T cells, and highlight a key role of TGF-β1 in the up-regulation of co-inhibitory molecules in tumor immunosuppression.

## RESULTS

### Co-expression of TGF-β1, B7-H3 and B7-H4 in colorectal cancer

**Figure 1:**
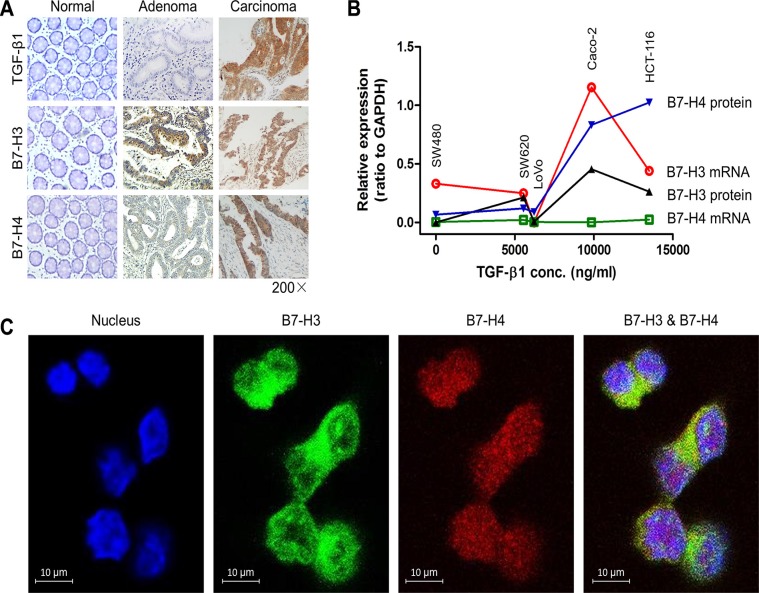
The expression of TGF-β1, B7-H3 and B7-H4 in colorectal cancer (**A**) The expression of B7-H3 and B7-H4 in normal, adenoma, and carcinoma colon tissues. (**B**) The positive relationship between the levels of autocrine TGF-β1 in media and the expression of B7-H3 and B7-H4 in CRC cells. (**C**) The immunofluorescent results showed co-expression of B7-H3 and B7-H4 on HCT-116 cells.

To investigate the relationship between TGF-β1 and co-inhibitory molecules B7-H3 and B7-H4 in colorectal cancer (CRC), we first measured the expression levels of TGF-β1, B7-H3 and B7-H4 in 78 CRC tissues and 5 cell lines. We found co-expression of TGF-β1, B7-H3 and B7-H4 in both CRC tissues (Figure 1A) and cell lines (Figure 1B). Furthermore, we found that the expression of autocrine TGF-β1 was positively correlated to those of B7-H3 and B7-H4 in both CRC tissues (Table 1) and cell lines (Figure 1B), indicating a potential interaction between them. Co-expression of B7-H3 and B7-H4 on colorectal cancer cells were also confirmed by using immunofluorescent analysis (Figure 1C).

**Table 1:** The association of the expression of TGF-β1 with the expression of B7-H3 and B7-H4 in colorectal cancer tissues

		TGF-β1		OR (95%CI)	*P* value
		Positive *n* (%)	Negative *n* (%)		
B7-H3	Positive	28 (35.90)	21 (26.92)	2.96 (1.12–7.81)	0.035
	Negative	9 (11.54)	20 (25.64)		
B7-H4	Positive	30 (38.46)	20 (25.64)	4.50 (1.61–12.5)	0.004
	Negative	7 (8.97)	21 (26.92)		

### TGF-β1 induced the expression of B7-H3 and B7-H4 in colorectal cancer cells

**Figure 2:**
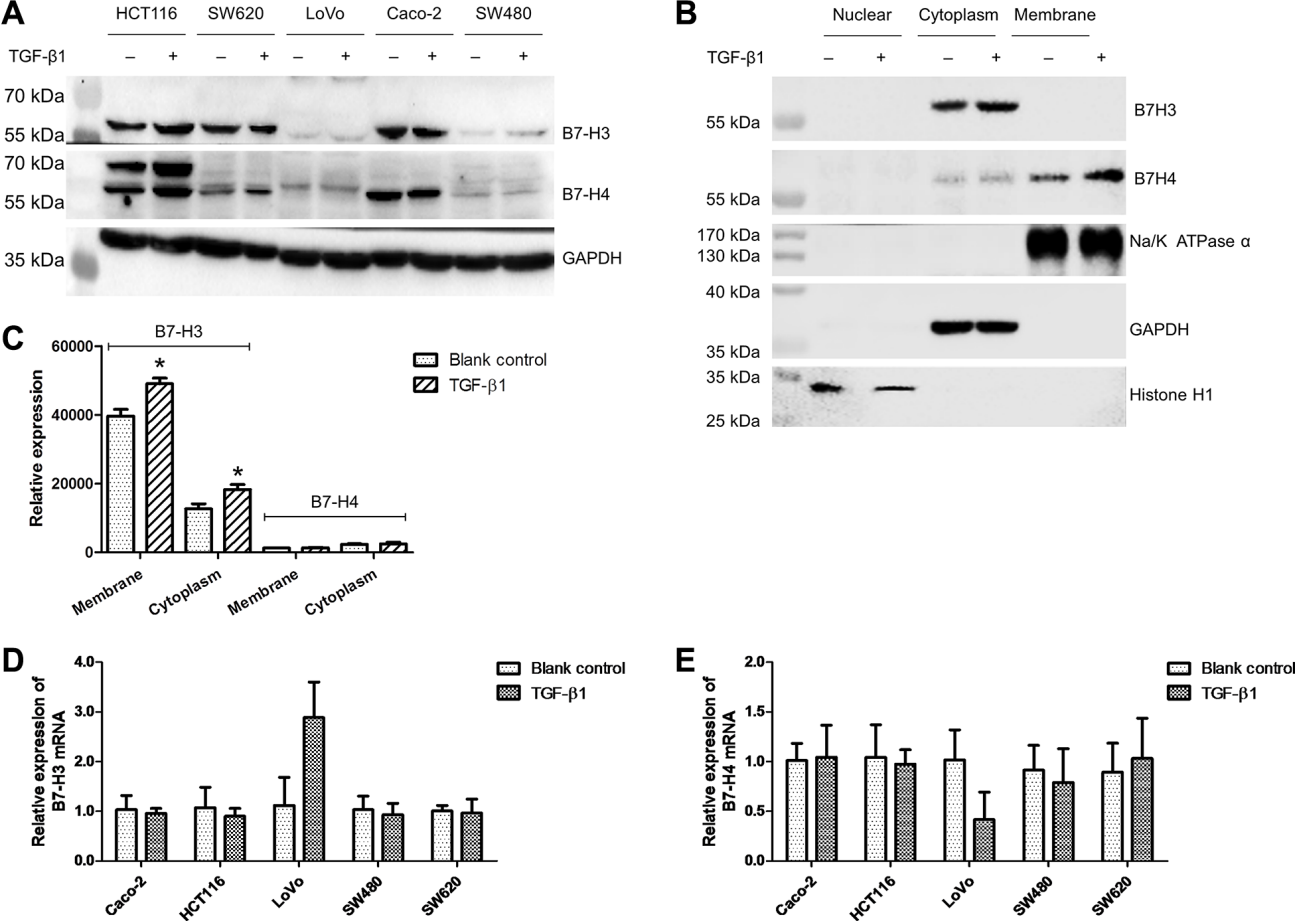
TGF-β1 elevated the expression of B7-H3 and B7-H4 in colorectal cancer cells (**A**) The western blot results showing the impact of TGF-β1 on the expression of B7-H3 and B7-H4 in the whole cell lysates of five CRC cell lines. (**B**) The western blot results showing the impact of TGF-β1 on the expression of B7-H3 and B7-H4 in the nucleus, cytoplasm, and membrane of HCT-116 cells. (**C**) The flow cytometry results showing the impact of TGF-β1 on the expression of B7-H3 and B7-H4 in the cytoplasm and membrane of HCT-116 cells. (**D**) The impact of TGF-β1 on B7-H3 mRNA expression in five CRC cell lines. (**E**) The impact of TGF-β1 on B7-H4 mRNA expression in five CRC cell lines. *t-test; *P* < 0.05.

To test the effect of TGF-β1 on the expression of B7-H3 and B7-H4 in CRC cells, we treated HCT-116, LoVo, Caco-2, SW480, and SW620 cells, with recombinant human TGF-β1. We found that TGF-β1 significantly elevated the expression of B7-H3 and B7-H4 in the whole cell lysates of HCT-116, SW480, and SW620 cells (Figure 2A). Since HCT-116 cells have high expression of B7-H3 and B7-H4, we chose them to investigate the signal pathway of TGF-β1 in the following experiments. Then we investigated the influence of TGF-β1 on the subcellular expression of B7-H3 and B7-H4 in HCT-116 cells, and found augmented B7-H3 expression in the cytoplasm and elevated B7-H4 expression in both the cytoplasm and the membranes (Figure 2B) by using western blotting analysis. We did not observe B7-H3 expression on the membranes of HCT-116 cells (Figure 2B), possibly because the antibody could not bind with B7-H3 protein on the membrane of HCT-116 cells. The flow cytometry results demonstrated that TGF-β1 evidently increased the B7-H3 expression not only on the membrane but also in the cytoplasm (Figure 2C). However, TGF-β1 did not alter the expression of B7-H3 and B7-H4 mRNA in CRC cells, except for LoVo cells in which there were no or low expression of B7-H3 and B7-H4 proteins (Figure 2D and 2E). These data suggest that the expression of B7-H3 and B7-H4 proteins on CRC cells might be post-transcriptionally elevated through a pathway mediated by TGF-β1.

### Dysregulated miRNAs in CRC

**Figure 3:**
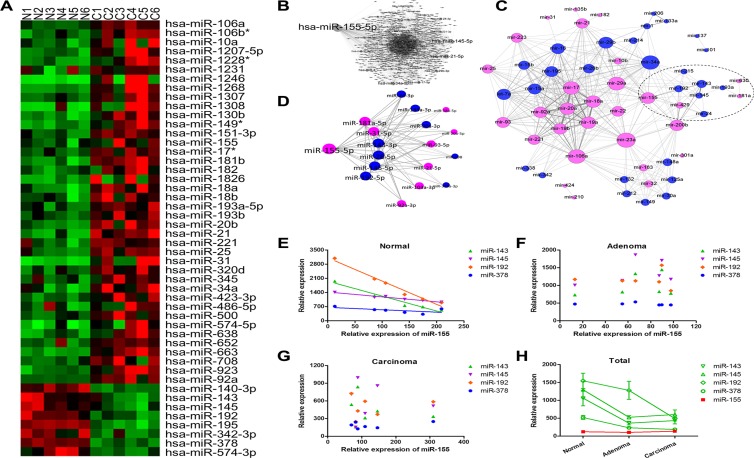
The expression, functional analysis, and inter-relationship of the dysregulated miRNAs in adenoma and carcinoma tissues (**A**) MiRNA array results showing 40 upregulated and 8 downregulated miRNAs in CRC tissues. (**B**) miRNA-miRNA functional synergistic network by assembling all miRNA synergistic pairs based on the miRNA target genes. (**C**) MiRNA-miRNA functional synergistic network by assembling all miRNA synergistic pairs based on the transcription factors reported to regulate the miRNAs. (**D**) MiRNA-miRNA functional synergistic network by assembling all miRNA synergistic pairs based on the correlation coefficients between each two miRNAs. (**E**) Relationship between the expression of miR-155 and the expression of miR-143, miR-145, miR-192, and miR-378 in normal colon tissues. (**F**) Relationship between the expression of miR-155 and the expression of miR-143, mir-145, miR-192, and miR-378 in adenoma tissues. (**G**) Relationship between miR-155 expression and the expression of miR-143, miR-145, miR-192, and miR-378 in carcinoma tissues. (**H**) Mean expression of miR-143, miR-145, miR-155, miR-192, and miR-378 in normal, adenoma, and carcinoma tissues. Each dot in Figures (E, F, and G) represents the expression level of one miRNA in one tissue sample

Many studies have demonstrated that microRNAs (miRNAs) play a key role in the post-transcriptional regulation of gene expression [25, 26]. Thus, we hypothesize that B7-H3 and B7-H4 are post-transcriptionally upregulated by TGF-β1 through miRNAs. We measured the expression of miRNAs in colorectal tissues by miRNA array. We found 8 down-regulated miRNAs and 40 up-regulated miRNAs in the cancer tissues as compared with the normal tissues (Figure 3A). These results were consistent to those published before (http://mircancer.ecu.edu/index.jsp). We collected the target genes of these dysregulated miRNAs from an online database miRTarBase (Supplementary Table S1) and used Gephi to construct the miRNA-miRNA functional synergistic network by assembling all miRNA synergistic pairs, in which nodes represented synergistic miRNAs and edges represented the co-regulated pathways between two miRNAs. As showed in Figure 3B, the miR-155 node was the largest, suggesting that miR-155 might be one of the most important miRNAs in CRC. Additionally, we used Gephi to construct the miRNA-miRNA functional synergistic network by assembling all miRNA synergistic pairs based on the transcription factors that have been reported to regulate the miRNAs (Supplementary Table S2). We found that the miR-155 node was close to those of miR-143, miR-145, and miR-192, etc. (Figure 3C), implying a potential interaction among them. Interestingly, we found that the expression of miR-155 was inversely proportional to those of miR-143, miR-145, miR-192, and miR-378 in normal tissues (Figure 3D and 3E; Supplementary Table S3), conversely to the expression in adenoma and carcinoma tissues (Figure 3F and 3G). In the adenoma tissues, we found that the expression of miR-155, miR-143, miR-145, miR-192, and miR-378 were initially deregulated (Figure 3H). Meanwhile, the expression of B7-H3 was also elevated in adenoma tissues (Figure 1A). To gain insight into the biological process that might involve these dysregulated miRNAs, KEGG pathway enrichment analysis was conducted by using ClueGo with the target genes of the dysregulated miRNAs (Supplementary Table S1). We found that miR-143 and miR-145 were mostly related to the TGF-β signaling pathway (Supplementary Table S4), suggesting a regulatory role of TGF-β1 in their expression.

### TGF-β1 increased miR-155 expression through SMAD3 and SMAD4

**Figure 4:**
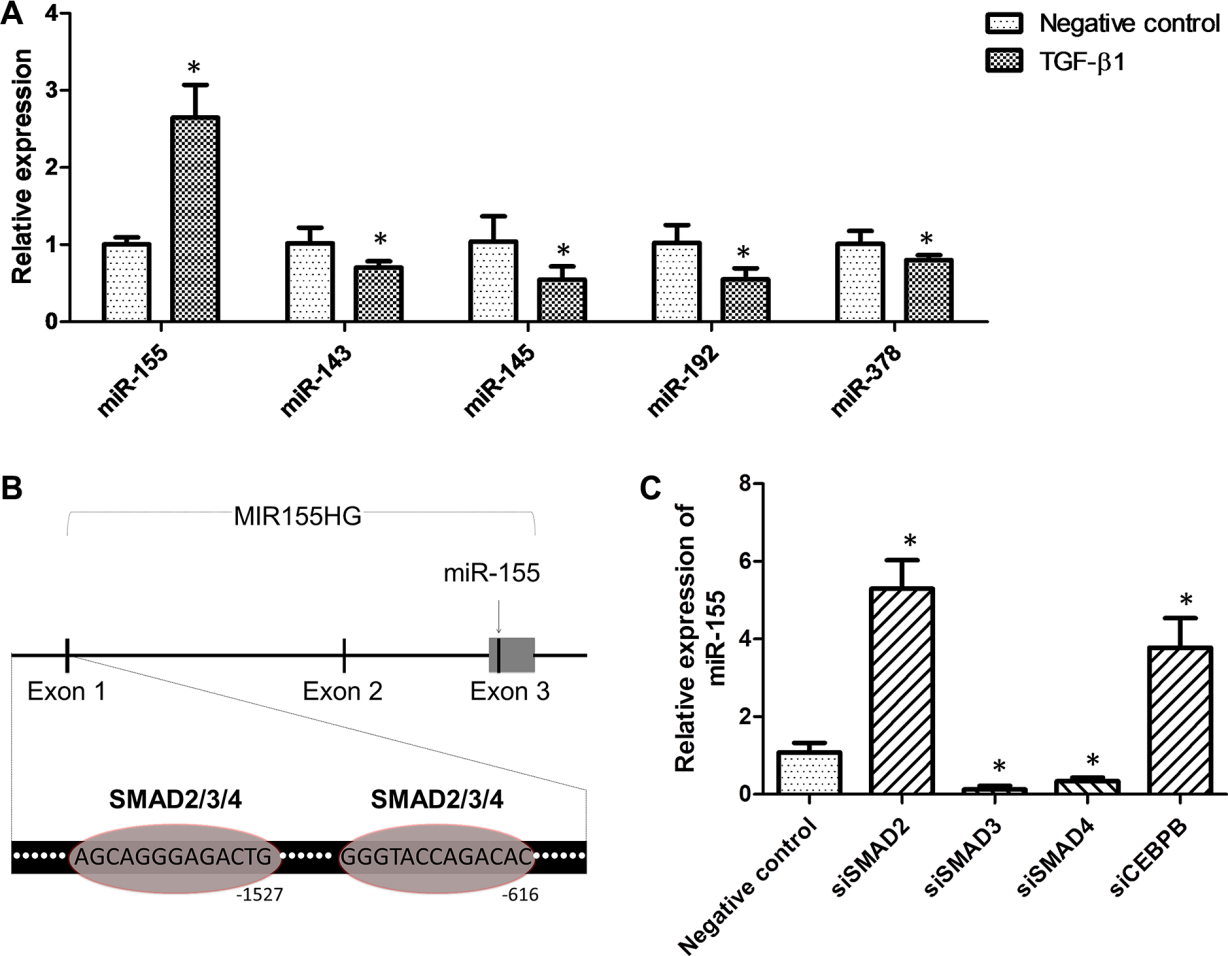
TGF-β1 elevated miR-155 expression in a SMAD3- and SMAD4-dependent pathway (**A**) Impact of TGF-β1 on the expression of miR-143, miR-145, miR-155, miR-192, and miR-378 in HCT-116 cells. (**B**) Predicted binding-sites of SMAD2/3/4 in the promoter of the MIR155HG gene. (**C**) Impact of transcription factors knockdown on miR-155 expression in HCT-116 cells. *t-test; *P* < 0.05.

To investigate whether or not the dysregulated miRNAs in CRC were regulated by TGF-β1, we measured the level of miRNAs expression in HCT-116 cells upon the treatment of TGF-β1. We found that TGF-β1 upregulated the expression of miR-155, while downregulating the expression of miR-143, miR-145, miR-192, and miR-378 (Figure 4A). Meanwhile, we found that the levels of autocrine TGF-β1 in the media were positively correlated to intracellular miR-155 expression in the cell lines (Supplementary Figure S1). Then, we used the online bioinformatics software JASPAR (http://jaspar.genereg.net/) to predict the potential binding sites of transcription factors on the promotor of miR-155 host gene (MIR155HG). As shown in Figure 4B, two binding-sites of SMAD2/3/4 were predicted to be in the MIR155HG promotor. When either SMAD3 or SMAD4 was silenced, the expression of miR-155 was evidently suppressed (Figure 4C). However, silencing of SMAD2 or CEBPB led to an elevated expression of miR-155 (Figure 4C). Since SMAD2 and CEBPB were discovered to be the direct targets of miR-155, there might be inverse feedback interactions between miR-155 and SMAD2 or CEBPB [27, 28].

### MiR-155 abated miR-143 expression through CEBPB

**Figure 5:**
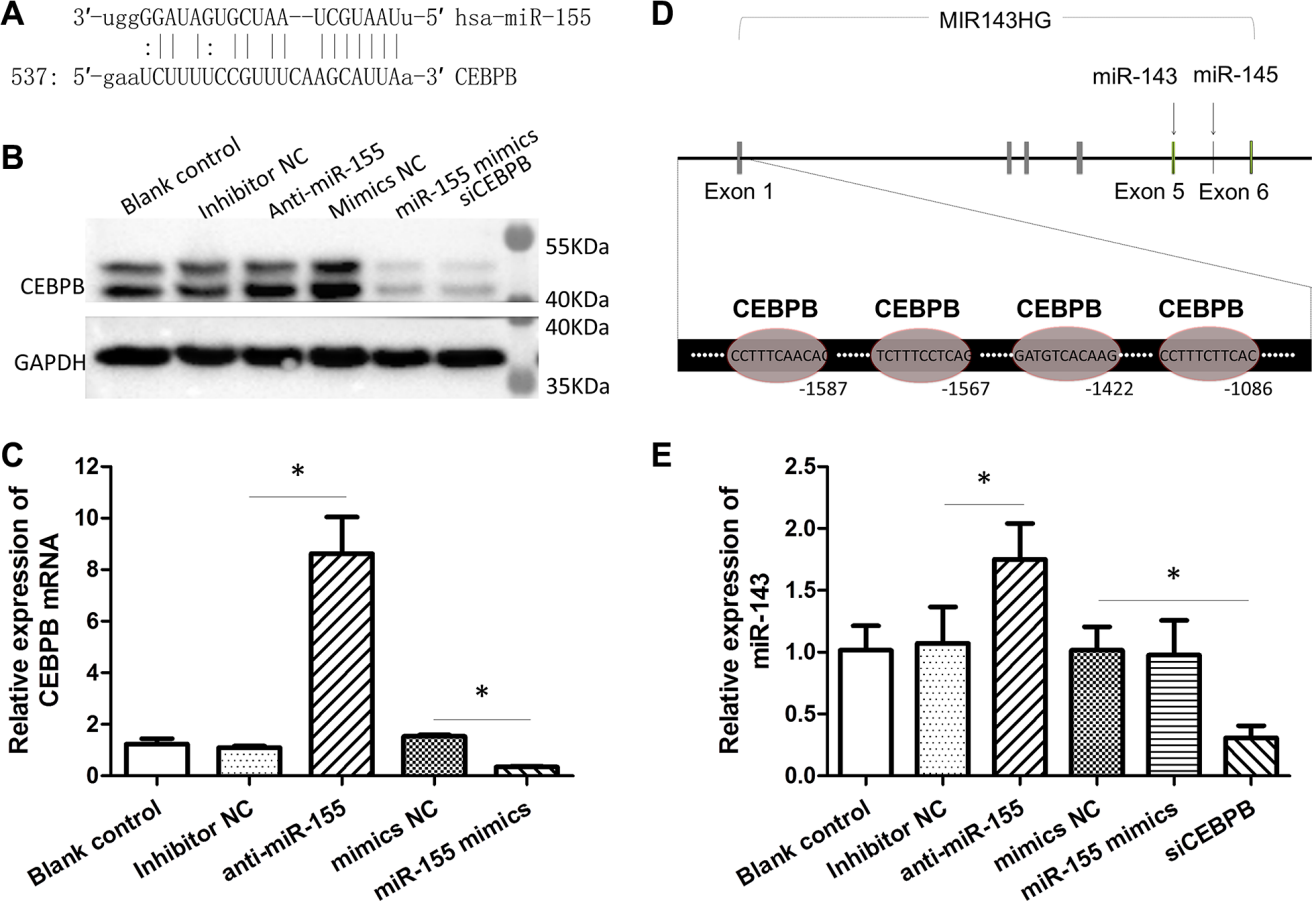
MiR-155 diminished the expression of miR-143 through CEBPB (**A**) Predicted binding-site of miR-155 in the 3′-UTRs of CEBPB gene. (**B**) Impact of the miR-155 inhibitor or mimics on the expression of CEBPB protein. CEBPB siRNA was used as positive control. (**C**) Impact of the miR-155 inhibitor or mimics on the expression of CEBPB mRNA. (**D**) Predicted binding-sites of CEBPB in the promoter of the MIR143HG gene. (**E**) Impact of the miR-155 inhibitor/mimics or CEBPB siRNA on the expression of miR-143. *t-test; *P* < 0.05.

The mir-143 has been reported to be repressed by miR-155 via targeting C/EBPβ (a transcriptional activator for mir-143) [29]. To further confirm that CEBPB is downregulated by miR-155 in CRC, we first predicted the interaction between miR-155 and CEBPB using the bioinformatics softwares miRanda and TargetScan. As showed in Figure 5A, there is a possible binding site for miR-155 in the 3′-UTR of the CEBPB gene. Both gain and loss of function assays confirmed that miR-155 obviously repressed the expression of CEBPB protein and mRNA in the HCT-116 cells (Figure 5B and 5C). Next, we found that there were four potential binding sites for CEBPB in the promoter of miR-143 host gene (MIR143HG; Figure 5D). The inhibition of CEBPB resulted in an attenuation of miR-143, indicating a regulatory role for CEBPB in the expression of miR-143 (Figure 5E). Additionally, the repression of miR-155 increased the expression of miR-143 (Figure 5E). Besides, we found that the expression of miR-143 was negatively correlated to miR-155 in CRC cell lines (Supplementary Figure S2).

### miR-143 inhibited the expression of B7-H3 and B7-H4 in CRC cells

**Figure 6:**
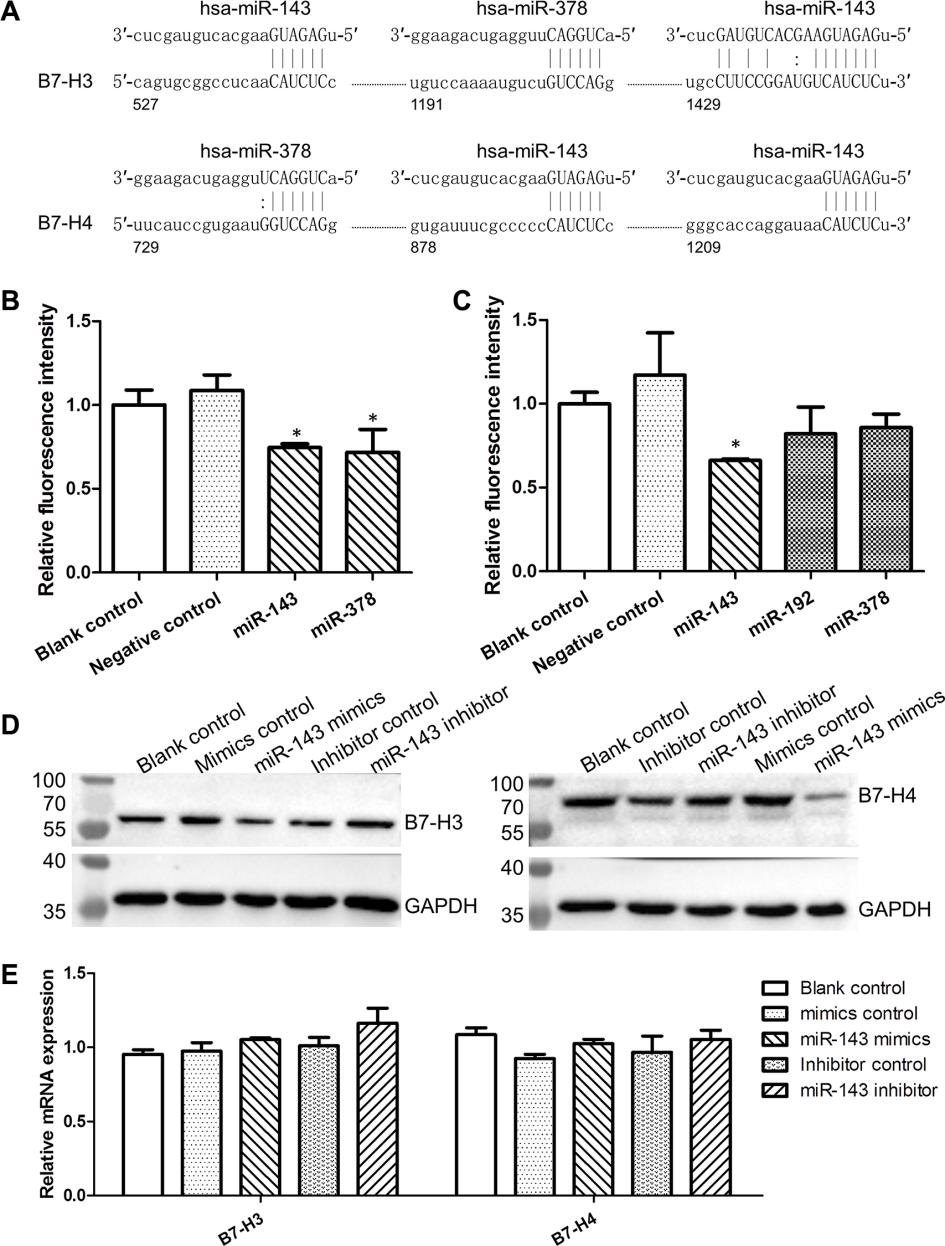
MiR-143 inhibited the expression of B7-H3 and B7-H4 (**A**) Predicted binding-sites of miR-143 and miR-378 in the 3′-UTRs of B7-H3 and B7-H4 genes. (**B**) Luciferase reporter analysis of the impact of miRNAs on the expression of the B7-H3/3′-UTR/pGL3 constructs. (**C**) Luciferase reporter analysis of the impact of miRNAs on the expression of the B7-H4/3′-UTR/pGL3 constructs. (**D**) Impact of miR-143 on the expression of B7-H3 and B7-H4 proteins. (**E**) Impact of miR-143 on the expression of B7-H3 and B7-H4 mRNAs. *t-test; *P* < 0.05.

To investigate the regulatory role of the deregulated miRNAs in the expression of co-inhibitory B7/CD28 molecules, we first predicted the binding-sites of miRNAs within the 3′-UTRs of B7/CD28 genes using the bioinformatics softwares miRanda and TargetScan (Figure 6A and Supplementary Figure S3A). Then, we constructed pGL3 luciferase reporter plasmids containing the 3′-UTRs of B7/CD28 genes and co-transfected them with miRNA mimics in CHO cells. We found that miR-143 significantly inhibited the expression of the B7-H3/3′-UTR/pGL3 and B7-H4/3′-UTR/pGL3 constructs (Figure 6B and 6C). The miR-143 mimics also repressed the expression of B7-H3 and B7-H4 proteins in HCT-116 cells and the expression was restored by miR-143 inhibitors (Figure 6D). However, neither miR-143 mimics nor miR-143 inhibitors impacted the expression of B7-H3 and B7-H4 mRNAs in HCT-116 cells (Figure 6E). Furthermore, we found that the expression of B7-H3 and B7-H4 proteins were negatively correlated with miR-143 in CRC cell lines (Supplementary Figure S4). We also found that miR-143 apparently suppressed the expression of the CTLA4/3′-UTR/pGL3 and B7-DC/3′-UTR/pGL3 constructs (Supplementary Figure S3). In addition, our results showed that miR-145, miR-192, and miR-378 suppressed the expression of co-inhibitory molecules B7-H3, B7-DC, CTLA4, and/or PD-1 (Supplementary Figure S3). These findings indicate that the lowly expressed miRNAs, especially miR-143, contributed a lot to the over-expression of co-inhibitory molecules in CRC, which consequently led to cancer immune evasion.

### B7-H3 and B7-H4 induced cytokines secretion by T cells to maintain a tumor immunosuppression microenvironment

**Figure 7:**
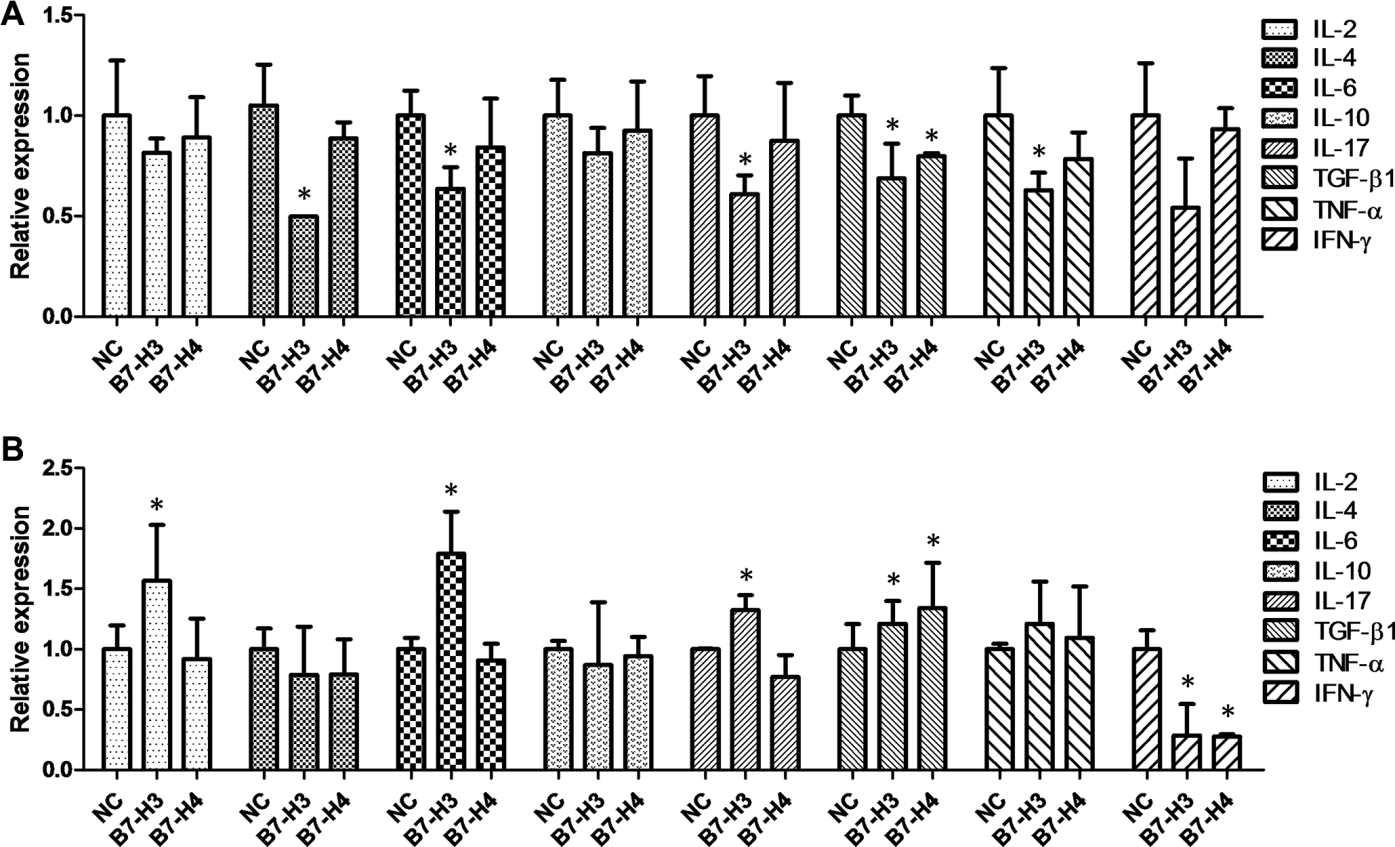
The effect of over-expression of B7-H3 and B7-H4 on the levels of autocrine cytokines in the media for HCT- 116 cells (A) or HCT-116 cells co-cultured with Jurkat cells (B)

A relatively high expression level of TGF-β1 at 13.50 ng/ml was detected in the HCT-116 cell culture, indicating that the HCT-116 self-secreted TGF-β1 to maintain a microenvironment facilitating tumor growth [30]. To explore the effect of B7-H3 and B7-H4 on the secretion of cytokines, we firstly upregulated B7-H3 and B7-H4 on HCT-116 cells and determined the expression level of autocrine cytokines in the media. We found that upregulation of B7-H3 obviously decreased the levels of IL-4, IL-6, IL-17, TGF-β1, and TNF-α, while upregulation of B7-H4 slightly attenuated the level of TGF-β1 (Figure 7A), suggesting a negative feedback effect of B7-H3 and B7-H4 on the expression levels of autocrine cytokines in HCT-116 cells.

Then we upregulated B7-H3 and B7-H4 on HCT-116 cells and co-cultured them with T-cell-derived Jurkat cells. We found that the upregulation of B7-H3 on HCT-116 cells apparently elevated the levels of IL-2, IL-6, IL-17, and TGF-β1 in the media (Figure 7B), and the upregulation of B7-H4 slightly increased the level of TGF-β1 in the media (Figure 7B). However, upregulation of either B7-H3 or B7-H4 abated the level of IFN-γ in the media (Figure 7B). These findings implied that the over-expression of B7-H3 and B7-H4 in CRC cells induced T lymphocyte cells to secrete immunosuppressive cytokines IL-2, IL-6, IL-17, and TGF-β1 to maintain a tumor microenvironment.

### MiR-143 inhibited the growth of CRC cells *in vitro* and *in vivo*

**Figure 8:**
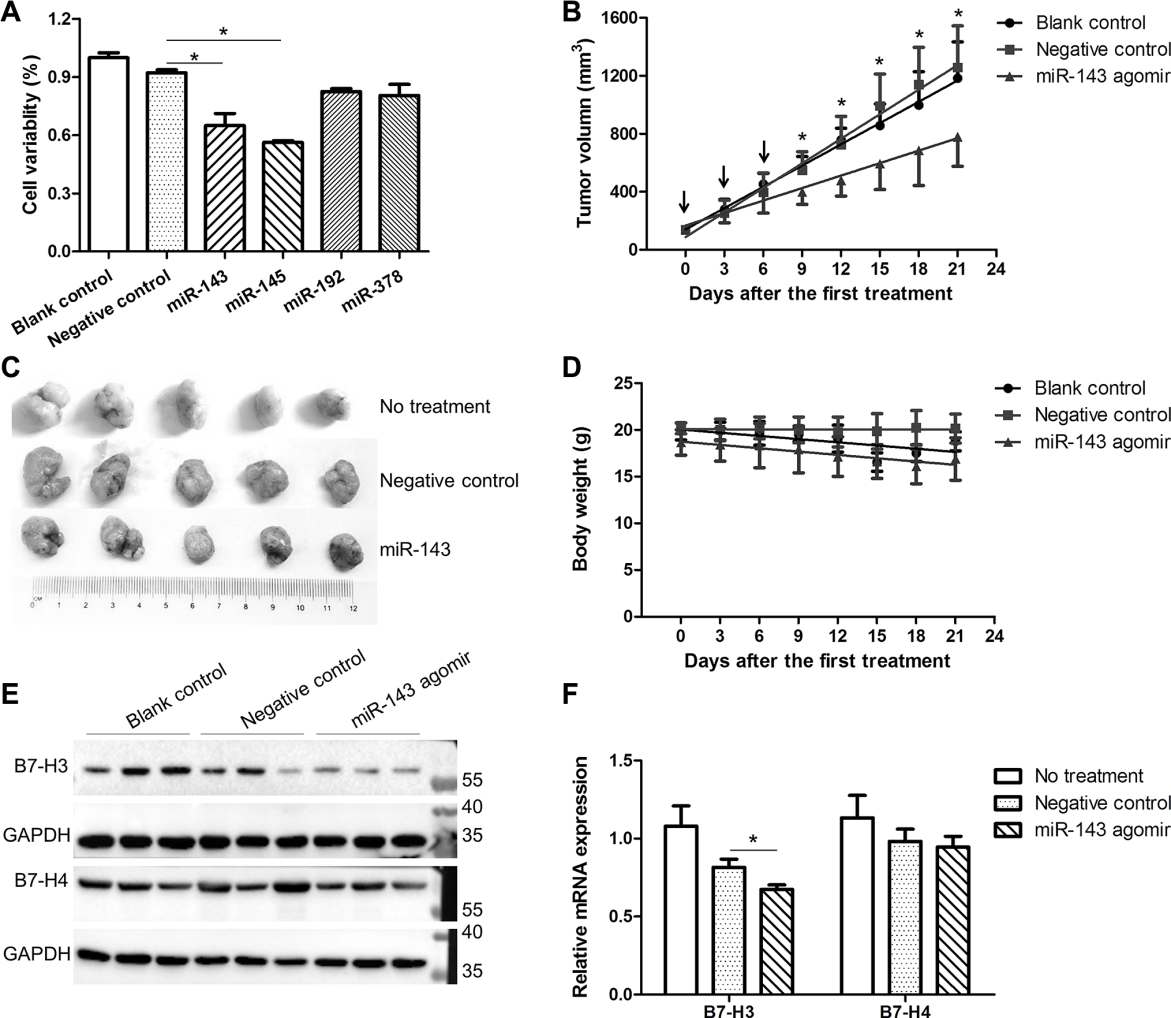
Effect of miRNAs on the growth of HCT-116 cells (**A**) Impact of miR-143, miR-145, miR-192, and miR-378 mimics on the growth of HCT-116 cells *in vitro*. (**B**) Impact of miR-143 agomir on the growth of HCT-116 xenograft tumors in nude mice (*n* = 5). The arrows indicate the intratumor treatments of miR-143 agomir at a dose of 2 nmol every three days for three times. (**C**) Pictures of tumors at 15 days after the last treatment. (**D**) Weight profile of mice. (**E**) Expression of B7-H3 and B7-H4 proteins in the xenograft tumors. (**F**) Expression of B7-H3 and B7-H4 mRNAs in the xenograft tumors. *t-test; *P* < 0.05.

Given the important role of down-regulated miRNAs in cancer immune escaping, we assessed the impact of miR-143, miR-145, miR-192, and miR-378 on the growth of HCT-116 cancer cells. The miR-143 and miR-145 mimics significantly inhibited the proliferation of the HCT-116 cells *in vitro* as compared with the scramble miRNA control (Figure 8A). Since miR-143 plays an important role in suppressing the expression of co-inhibitory molecules, we then evaluated the inhibitory role of miR-143 agomir on the tumor growth in mice. We subcutaneously grafted HCT-116 cells into the lower back of nude mice and let them grow until palpable tumors formed. On day 14 after xenograft implantation, a group of tumor-bearing mice received intratumoral (i.t.) injections of 1 mg/kg/day of miR-143 agomir or the negative control. Local injections were repeated on days 17 and 20 to maintain increased levels of miR-143 in the tumor tissues. As shown in Figure 8B and 8C, the tumors injected with negative control were unaffected and developed at a pace similar to those that received water. In contrast, i.t. injections of miR-143 agomir prevented the outgrowth of viable tumors (Figure 8B and 8C). The data suggested that local administration of miR-143 agomir induced a specific inhibitory effect in tumor cells. Meanwhile, the miR-143 agomir did not have any effect on the mice body weights (Figure 8D), indicating that the miR-143 agomir was safe for anti-tumor therapy. Interestingly, the expression of both B7-H3 and B7-H4 in the xenograft tumors was markedly repressed even at 15 days after the last administration of miR-143 agomirs (Figure 8E and 8F).

## DISCUSSION

**Figure 9:**
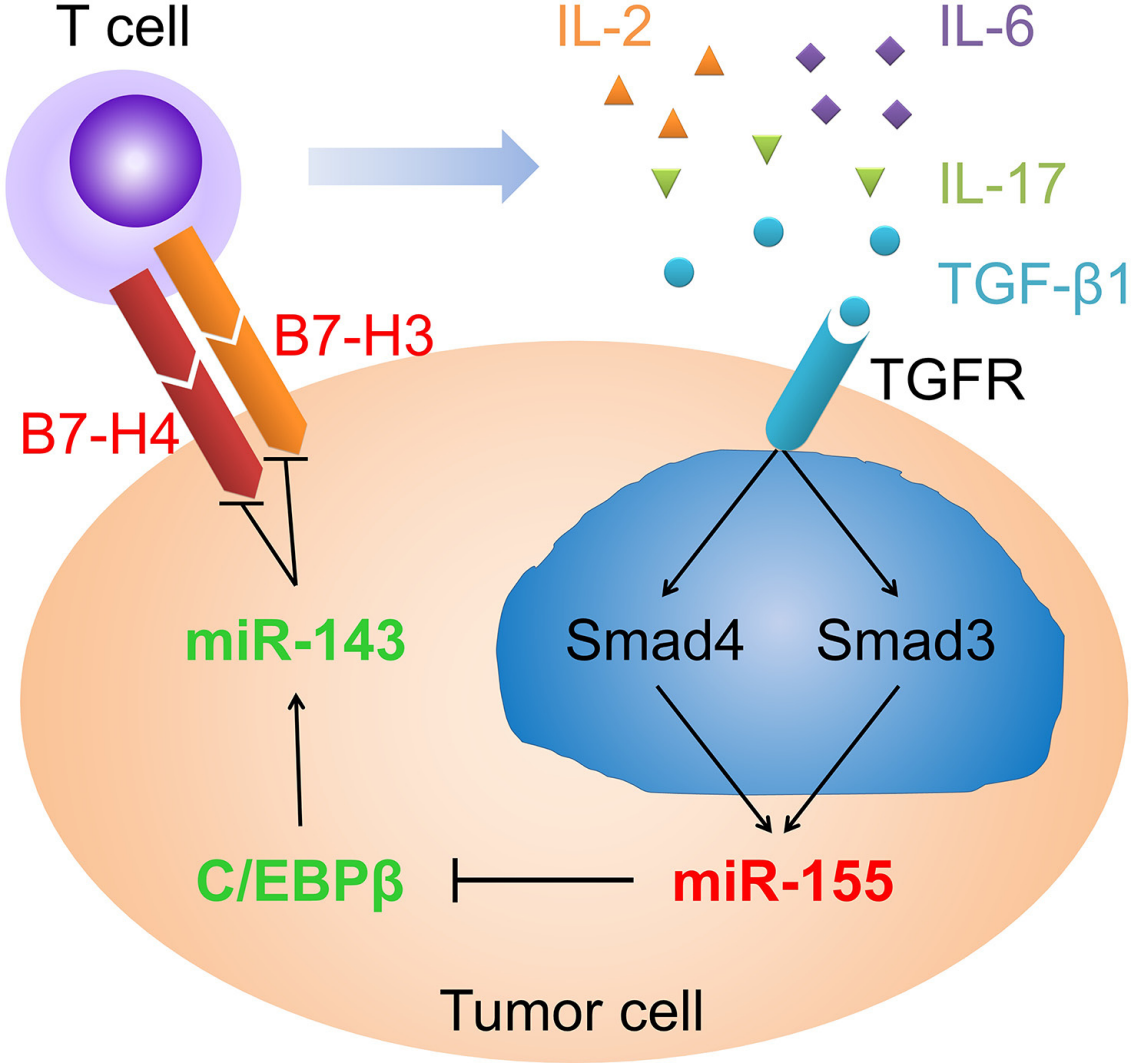
Schematic profile showing that TGF-β1 through SMAD3 and SMAD4 elevated miR-155 expression, which in turn attenuated the expression of CEBPB and consequently the expression of miR-143. The decrease in miR-143 disinhibited co-inhibitory molecules B7-H3 and B7-H4 on colorectal cancer cells In a feedback loop, the hoisted B7-H3 and B7-H4 promoted T cells to secrete immunosuppressive cytokines to maintain a tumor microenvironment.

High levels of TGF-β1 have been detected in either serum or tumor in various types of cancer. Although many efforts have been made to discover the regulatory roles of TGF-β1 in tumor progression, metastasis and angiogenesis [4], the mechanisms under immunosuppression in cancers are largely unknown. In this study, we provided evidence that TGF-β1 through SMAD3 and SMAD4 elevated miR-155 expression, which in turn attenuated CEBPB expression and consequently miR-143 expression. In turn, the decrease in miR-143 disinhibited co-inhibitory molecules B7-H3 and B7-H4 on colorectal cancer cells. In a feedback loop, the hoisted B7-H3 and B7-H4 promoted T cells to secrete immunosuppressive cytokines IL-2, IL-6, IL-17, and TGF-β1, to maintain a tumor microenvironment (Figure 9). Administration of miR-143 agomir attenuated B7-H3 and B7-H4 expression, resulting in inhibited growth of xenograft tumors in mice.

Previously, we observed strong B7-H3 positivity in colorectal carcinomas which correlated with tumor grade and decreased T lymphocyte cells. Notably, TNF-α, an inflammatory pro-tumorigenic molecule, could induce the shedding of soluble B7-H3 by CRC cells, suggesting another mechanism of cancer immune evasion via B7-H3 [31]. Tumor-associated macrophages were observed to upregulate the expression of B7-H4 through IL-6 and IL-10 in the tumor microenvironment [32]. These findings indicate that B7-H3 and B7-H4 can be induced by cytokines in the tumor microenvironment. Here, we found that TGF-β1 increased the expression of B7-H3 and B7-H4 in the cytoplasm and membrane of CRC cells. Furthermore, we provided evidence to clarify the regulatory mechanism of TGF-β1 through the miR-155/miR-143 axis in a SMAD3/4-dependent pathway. These findings expand our understanding of the regulatory role of cytokines, especially TGF-β1, in colorectal cancer immune evasion.

MiRNAs are increasingly recognized as important elements in the development and function of the immune system. Changes in miRNA expression profile are described in many diseases including cancers [33]. Compelling evidence indicates that miR-155 over-expresses in cancers and regulates cellular functions including development, proliferation, differentiation, metastasis, and chemosensitivity [34, 35]. In line with previous reports for the lymphoma cell lines [36] and mammary gland adenocarcinoma cells [37], we found that TGF-β1 positively regulates miR-155 expression in CRC cells. Altogether, these data indicate that the sustained increase in the levels of TGF-β1 in CRC patients can drive miR-155 overexpression in the cancer cells. Thus, taking into account the immunosuppressive roles of TGF-β1, a miR-155-mediated mechanism to escape immune surveillance may be present in CRC.

Down-regulation of miR-143, miR-145, miR-192, and miR-378 has also been well documented in many types of human tumors including colorectal cancer [38–40]. They contribute a lot to tumor initiation and development by controlling the expression and function of tumor suppressor genes, including K-RAS, ELK1, MYO6, BCL2, ERK5 and IGF1R [40–44]. In this study, the co-inhibitory molecules B7-H3 and B7-H4 have been experimentally validated as the novel targets of miR-143 *in vitro* and *in vivo*. Firstly, we confirmed by luciferase reporter assay that miR-143 directly recognize the 3′-UTR of B7-H3 and B7-H4 transcripts. Secondly, the expression of B7-H3 and B7-H4 was significantly abolished in CRC cells with stable expression of miR-143 and was increased in CRC cells with miR-143 silence. Thirdly, we found an inverse correlation between B7-H3 and B7-H4 proteins and miR-143 in CRC cells (Supplementary Figure S4). Fourthly, the expression of B7-H3 and B7-H4 in xenograft tumors was heavily repressed even at 15 days after the last treatment with miR-143 agomirs. Finally, the inhibition of B7-H3 and B7-H4 expression by miR-143 inhibited tumor growth in mice. In addition, we also provided evidence to support that miR-143 disinhibited the expression of B7-DC, CTLA4 and PD-1, thereby facilitating tumor immune escaping (Supplementary Figure S3). Furthermore, miR-145, miR-192, and miR-378 also showed inhibitory role in the expression of B7-DC, CTLA4 and PD-1. Taken together, our present study provides the first evidence that under-regulated miRNAs play significant roles in inhibiting translation of B7 co-inhibitory molecules, especially miR-143 in B7-H3 and B7-H4.

Numerous studies have explored the role of an increased expression of miR-143/145 in epithelial malignancies. Ectopic expression of miR-143/145 can actively deregulate signaling pathways through the Ras-Raf-MEK-ERK (MAPK cascade) in addition to modulating other proliferative signaling networks such as the PI3K-AKT pathway, TGF-β signaling via targeting TGF-β activated kinase (TAK1, MAP3K7) and Jun-N-terminal Kinase (JNK) [45]. The induction of miR-143/145 expression in various epithelial cancers such as colon, gastric, cervical, pancreatic carcinomas and adenocarcinomas blocked tumorigenesis both *in vitro* and *in vivo* [46, 47]. Enforced expression of miR-143/145 via systemic intravenous delivery with nanoparticles has been shown to inhibit tumor growth of orthotopic pancreatic xenografts by downregulating K-RAS and RREB-1, respectively [48]. This present study is the first to show that miR-143 suppresses colorectal xenografts tumor growth by diminishing the expression of B7-H3 and B7-H4. Therefore, the role of miR-143/145 in the tumor suppression cannot be ignored, and collectively, these studies highlight the potential use for miR-143 to target genes or networks commonly dysregulated in epithelial cancers.

Similar to the colorectal cancer tissues, individual differences were observed in the expression of B7-H3 and B7-H4 proteins in cancer cell lines (Figure 1A). This might because that the cancer cell lines are derived from different patients with different genetic backgrounds. For instance, the expression levels of miR-143 are quite different among these cell lines (Supplementary Figure S4). When we tested the effect of TGF-β1 on the expression of mRNA and protein of B7-H3 and B7-H4, we found that the effects of TGF-β1 on the expression of mRNA and protein of B7-H3 and B7-H4 were almost the same among CRC cell lines (Figure 2A, 2D and 2E). However, differences in the effects of TGF-β1 were also observed. TGF-β1 apparently elevated the expression of B7-H3 protein but had no effect on the expression of B7-H4 protein in SW480 cells (Figure 2A). In LoVo cells, TGF-β1 elevated the expression of B7-H3 mRNA but downregulated the expression of B7-H4 mRNA (Figure 2D and 2E). This may also be due to the different genetic backgrounds of the cancer cell lines, such as the expression levels of downstream molecules (TGF-β1 receptors and/or SMADs, etc.) in the TGF-β1 pathway [49]. Meanwhile, the differences in the effects of miRNAs on the expression of B7-H3 and B7-H4 were also observed in this study. The results from Figure 6B–6E demonstrate that miR-143 can inhibit the expression of B7-H3 and B7-H4 proteins to the same extent. However, the data in Figure 8E–8F indicate that miR-143 can only suppress the expression of B7-H3 protein *in vivo*. One possible reason for this might be the difference between *in vivo* microenvironment and *in vitro* microenvironment.

In summary, we discovered new regulatory mechanisms for the intensified expression of co-inhibitory B7/CD28 family molecules in colorectal cancer mediated by TGF-β1, which deregulates miRNAs, likely contributing to the immune escape of colorectal cancer. These findings may also offer a potential new therapeutic strategy for treating colorectal cancer. Taken together with previous reports showing that abnormal expression of B7/CD28 family molecules are regulated by miRNAs other than those revealed in this study [18–24], our results imply that multiple mechanisms may contribute to the expression of these molecules in cancers and these regulatory mechanisms may be different among individuals, especially microenvironments. Thus, the regulatory mechanisms modulating the expression of B7/CD28 family molecules, other than those reported here need to be further investigated. Therefore, a precise understanding of the mechanisms by which TGF-β1 alters co-inhibitory molecules through miRNAs and their specific involvement in tumor immune evasion may improve cancer immunotherapies.

## MATERIALS AND METHODS

### Tissue samples

The Institutional Review Board of Soochow University approved this study. After written informed consent, 78 pairs of fresh tissue samples including cancer and normal tissue samples (> 5 cm away from the cancer margin) and 6 adenoma tissues were collected from participants, for detecting the expression of miRNAs, mRNAs, and proteins. Two pathologists histologically confirmed the patients' samples. None of the patients had undergone radiotherapy or chemotherapy before surgery. All subjects were genetically unrelated ethnic Han Chinese and were born and raised in Wuxi, China.

### Cell culture

The Caco-2, HCT-116, LoVo, Jurkat, SW480, SW620, and CHO cells were purchased from American Type Culture Collection (Manassas, VA). They were cultured in DMEM or RPMI 1640 medium (Hyclone) containing 10% fetal bovine serum (FBS; GIBCO) at 37°C in 5% CO_2_. Cells in the logarithmic growth phase were used for experiments.

Cell cultures were treated with 10 ng/ml of TGF-β1 (R&D Systems) to investigate its effect on the expression of miRNAs, mRNAs, and proteins.

### Gene silencing

Fifty nM synthetic RNAs including siSMAD2, siSMAD3, siSMAD4, siCEBPB, siCYMC, miR-155 mimics/inhibitor, and miR-143 mimics/inhibitor (GenePharma, Shanghai, China; see Supplementary Table S5), were transfected into HCT-116 cells with lipofectamine 2000 (Invitrogen) for 72 h before the experimental treatments.

### MicroRNA array

A total of 830 human miRNAs were determined in six pairs of normal, adenoma, and carcinoma tissues by using miRNA array analysis (Affymetrix). Relative expression was calculated using the fluorescence intensities provided by the manufacturer.

### Construction of miRNA-miRNA functional synergistic network

KEGG pathway functional module identification was performed by using the clusterProfiler package (http://www.bioconductor.org/) with the deregulated miRNAs and their verified targets (obtained from miRTarBase; http://mirtarbase.mbc.nctu.edu.tw/; Supplementary Table S1) or transcriptional factors (TransmiR; http://cmbi.bjmu.edu.cn/transmir; Supplementary Table S2). *P* < 0.05 was set as the threshold. MiRNAs which had the same target genes were considered to be synergistic miRNAs. A miRNA-miRNA functional synergistic network was constructed by assembling all synergistic miRNAs using Gephi (http://gephi.org/), in which nodes represented synergistic miRNAs and edges represented the pathways co-regulated between two miRNAs.

### KEGG pathway enrichment analysis

To further understand the biological relevance of the predicted genes of dysregulated miRNAs in colorectal cancer (CRC), a KEGG functional enrichment analysis was performed by using ClueGO (http://apps.cytoscape.org/apps/cluego), a plugin for Cytoscape, which could visualize the selected terms in a functionally grouped annotation network to reflect the relationships between terms based on the similarity of their associated genes. Here, the statistical test used for enrichment was based on right-sided hypergeometric option with a Bonferroni step-down correction and a kappa score of 0.3. Only terms with corrected *p-value* ≤0.05 were considered, and the annotation network was then laid out by Cytoscape 3.1.0.

### Luciferase reporter assay

The luciferase reporter assays were carried out as before [50]. Briefly, the binding-sites of miRNAs within the 3′-UTRs of target genes were predicted by using the bioinformatics softwares miRanda (http://www.microrna.org/) and TargetScan v5.1 (http://targetscan.org/). The 3′-UTRs of B7-H1, B7-DC, B7-H3, B7-H4, CTLA-4, and PD-1 genes were amplified from the cDNA samples with the primer pairs listed in Supplementary Table S6. They were then cloned into pGL3-control vector (Promega) by using *Xba*I and *Hpa*l or *Kpn*l (for B7-H1, B7-H3, and B7-H4) endonucleases (NEB). The positive clones were confirmed by using PCR, restriction enzymes digestion, and DNA sequencing methods (GenScript, Nanjing, China). The 3′-UTR/pGL3 constructs were co-transfected into CHO cells with miRNA mimics using lipofectamine 2000 (Invitrogen), in triplicate. The pRL-TK plasmid (Promega) was used as a normalizing control, and scramble miRNA mimics was used as negative control. The transfected CHO cells were grown in optimum medium (GIBCO) with 10% FBS. After 24 hours of incubation, the cells were collected and analyzed for the level of luciferase activity with the dual-luciferase reporter assay system (Promega).

### Quantitative real-time PCR

Total RNA was isolated from cancer tissues and Caco-2, HCT-116, LoVo, SW480, and SW620 cells by using TRIzol (Takara) according to the manufacturer's instructions and subjected to reverse transcription with NxGen M-MuLV reverse transcriptase (MBI) and stem-loop primers (Supplementary Table S6), or scramble primers (GeneWiz, Suzhou, China) for B7-H3, B7-H4, SMAD2, SMAD3, SMAD4, CEBPB, TP53, GAPDH and U6. Quantitative real-time PCR (qPCR) was conducted in 20 μl reaction containing 400 nM of each primer (Supplementary Table S6) and quantitative RT-PCR master mix (Bio-Rad). The real-time PCR was conducted on the CFX96 Touch^TM^ real-time PCR system (Bio-Rad). The relative expression was calculated by using the comparative threshold cycle (Ct) method with the formula 2^−ΔΔCt^. A Ct value ≥ 30 was interpreted as amplification too low to quantify.

### Immunohistochemistry

The paraffin-embedded samples were cut into 5-μm sections, which were then analyzed by using immunohistochemistry method. After deparaffinization in xylene, the sections were incubated with antibodies against human TGF-β1, B7-H3 and B7-H4 (Santa Cruz Biotech, USA). The adjacent sections were stained with diaminobenzidine or 3-amino-9-ethylcarbazole in an Envision System (Dako). The slides were viewed and imaged with a microscope system (Olympus). All slides were reviewed by two independent hematopathologists who were blinded with the clinical outcome.

### Cell lysates and cell fractionations

Total proteins were prepared by lysing the cells with RIPA buffer (Beyotime, Shanghai, China) supplemented with the complete protease inhibitor cocktail on ice for 30 min. Then the cell lysates were centrifuged at 4°C, 16000 g for 15 min.

To get the cytosolic fraction and membrane fraction, the cells were harvested and washed once with ice-cold PBS, then the cell pellets were re-suspended in homogenization buffer (0.32 mM sucrose, 20 mM HEPES, 0.5 mM EGTA, 5 mM NaN_3_ at pH 7.4) containing protease inhibitor mixture and homogenized using a glass homogenizer with a Teflon pestle. Homogenates were centrifuged at 1000 g for 10 min at 4°C to remove nuclei and any unbroken cells. Supernatants were then subjected to ultracentrifugation at 100,000 g for 1 hour at 4°C. The supernatants (cytosolic fraction) and pellets (membrane fraction) were collected, and their respective protein concentrations were determined.

To obtain nucleic fraction, the cells were washed and re-suspended in the same homogenization buffer as above, then homogenized using an RZR 2021 homogenizer (Heidolph Instruments, Germany) with 25 strokes at 1200 rpm. Three μl of nuclear suspension were placed in 3 ml of bath solution that contained 140 mM KCl, 10 mM HEPES, 500 μM 1,2-bis(2-aminophenoxy)ethane-N,N,N′,N′-tetraacetic acid, and 246 nM free Ca^2+^, pH 7.1. Nuclei were allowed to adhere to a plastic culture dish for 10 min.

### Western blot

Protein concentrations were determined using the Pierce BCA Protein Assay Kit (Thermo) according to the manufacturer's instructions. Twenty μg of protein were separated on 10% SDS-PAGE gel and electro-transferred onto PVDF membranes. The membranes were incubated with B7-H3, B7-H4, CEBPB, GAPDH, Histone H1 or Na/K ATPase rabbit polyclonal antibodies (Santa Cruz Biotech, USA) and subsequently with a peroxidase goat anti-rabbit IgG (Santa Cruz Biotech). The membranes were then developed with Clarity Western ECL substrates (Merck Millipore) and visualized with the ChemiDocTM MP Imaging System (Bio-Rad).

### Immunofluorescence

The plate (Thermo, US) was treated with 100 ug/mL polylysine (Sigma, US) for 15 minutes. HCT-116 cells were cultured on sterile glass slides overnight at 37°C. The slides were washed briefly with PBS. The cells on the slides were fixed by 4% polyformaldehyde in PBS for 20 minutes. The slides were washed for three times with PBS, and then were fronzed in pre-cooled 0.2% (W/V) Triton X-100 and blocked in 5% BSA. The slides were incubated overnight at 4°C with primary antibody goat anti-human B7-H3 (2 μg/ml) or rabbit anti-human B7-H4 (2 μg/ml). After washing, donkey anti-goat IgG-FITC or donkey anti-rabbit IgG-TR (Santa Cruz Biotech, USA) secondary antibody was added. As controls, the slides were counterstained with DAPI for nuclear localization. The sections were viewed using a Zeiss confocal microscope.

### Flow cytometry

Cells (~5 × 10^4^) were washed and stained for 20 min with murine antibodies against B7-H3 (clone MIH42, FITC), B7-H4 (clone MIH43, FITC), or FITC-labeled mouse IgG1 isotype controls (eBioscience). The stained cells were applied to the flow cytometer (Beckman Coulter) and 20,000 events were analyzed. Results of the cell staining are presented as histograms, with cell number on the vertical axis and relative fluorescence on the logarithmic horizontal axis. The levels of IL-2, IL-4, IL-6, IL-10, IL-17, IFN-γ, and TNF-α in the culture supernatants were detected with cytometric bead array (BD) and analyzed by using flow cytometry.

### ELISA

The TGF-β1 concentrations in the supernatants of HCT-116 cell cultures or HCT-116/Jurkat cell co-cultures were measured with an ELISA kit (R&D Systems). The supernatants were collected after 48 hours in culture. Before quantification, the supernatants were pre-treated with 1 M HCl and 1 M NaOH to activate the immunoreactive form, and then added in triplicate to appropriate plates coated with capture antibody. The plates were washed and then incubated with a horseradish peroxidase-conjugated detection antibody. The substrate used for color development was tetramethylbenzidine. The optical density was measured at 450 nm with a microplate reader (M3 SpectraMax microplate reader, Molecular Devices, USA). The experiments were independently repeated 3 times. The standard curve was determined by the recombinant TGF-β1 contained in the kit. The coefficient of variation for the linearity of the standard curve was < 2%.

### MTT assay

MTT assay was used to determine the effect of the down-regulated miRNAs, including miR-143, miR-145, miR-192, and miR-378, on the proliferation of HCT-116 cells. The HCT-116 cells were seeded in 96-well culture plates at 5000 cells/well. Twenty-four hours later, 50 nM of miRNAs were transfected into the cancer cells using lipofectamine 2000. Lipofectamine 2000 only or with scramble miRNA was used as blank control and negative control, respectively. The cells are incubated with MTT for 2 h (Sigma). Then the medium was replaced with 150 μl of dimethyl sulphoxide (Merck) and oscillated at 37°C for 10 min. We measured absorbance at 490 nm using the M3 SpectraMax microplate reader. The impact of miRNAs on cell growth was estimated according to the absorbance.

### Xenografts

All animal experiments were performed in accordance with currently prescribed guidelines and under a protocol approved by the Institutional Animal Care and Use Committee at Soochow University. HCT-116 cells were collected, counted, and mixed with Matrigel (BD Biosciences) in a 1:1 ratio by volume. Cells (5 × 10^6^) in 100 μL of medium/Matrigel solution were subcutaneously (s.c.) injected in the lower back region of male nude mice (SLAC int., Shanghai, China). Two nmol of miR-143 agomir was intratumorally (i.t.) administered 3 times, once every 3 days after the tumors have reached a volume of 150–200 mm^3^. Tumor volumes were measured every 3 days and were calculated by the formula (width^2^ × length/2).

## SUPPLEMENTARY MATERIAL FIGURES AND TABLES








