# Supplementary material for: TGF-β1 promotes colorectal cancer immune escape by elevating B7-H3 and B7-H4 via the miR-155/miR-143 axis
Source: Oncotarget. 2016 Sep 10;7(41):67196–211. doi: 10.18632/oncotarget.11950 (PMC5341868; doi:10.18632/oncotarget.11950)
Supplement: Supplementary file 1 [file oncotarget-07-67196-s001.pdf]

TGF-β1 promotes colorectal cancer immune escape by elevating B7-H3 and B7-H4 *via* the miR-155/miR-143 axis

Supplementary Materials

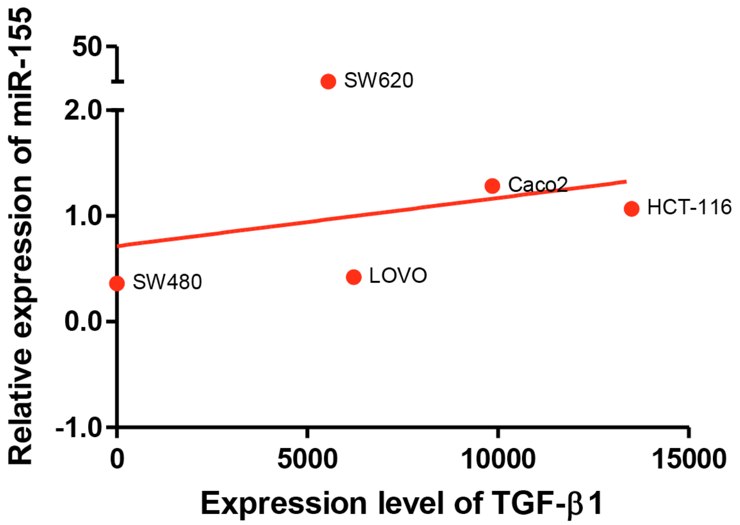

Supplementary Figure S1: The positive relationship between the expression level of TGF-β1 and the expression of miR-155 in CRC cell lines.

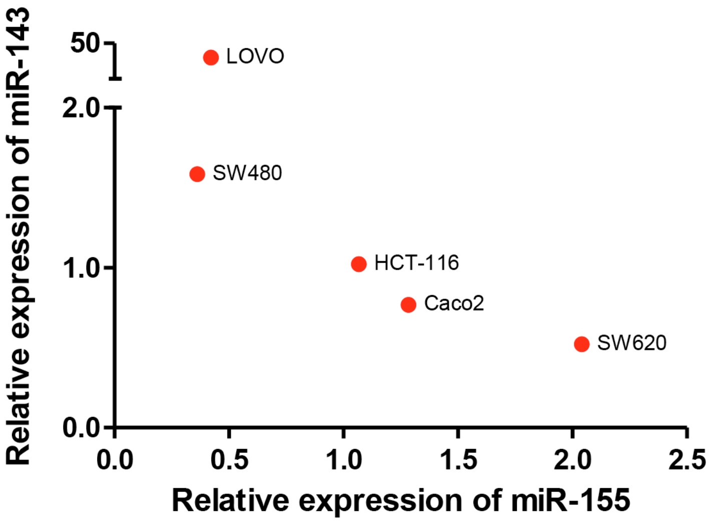

Supplementary Figure S2: The negative relationship between the expression of miR-155 and the expression of miR-143 in CRC cell lines.

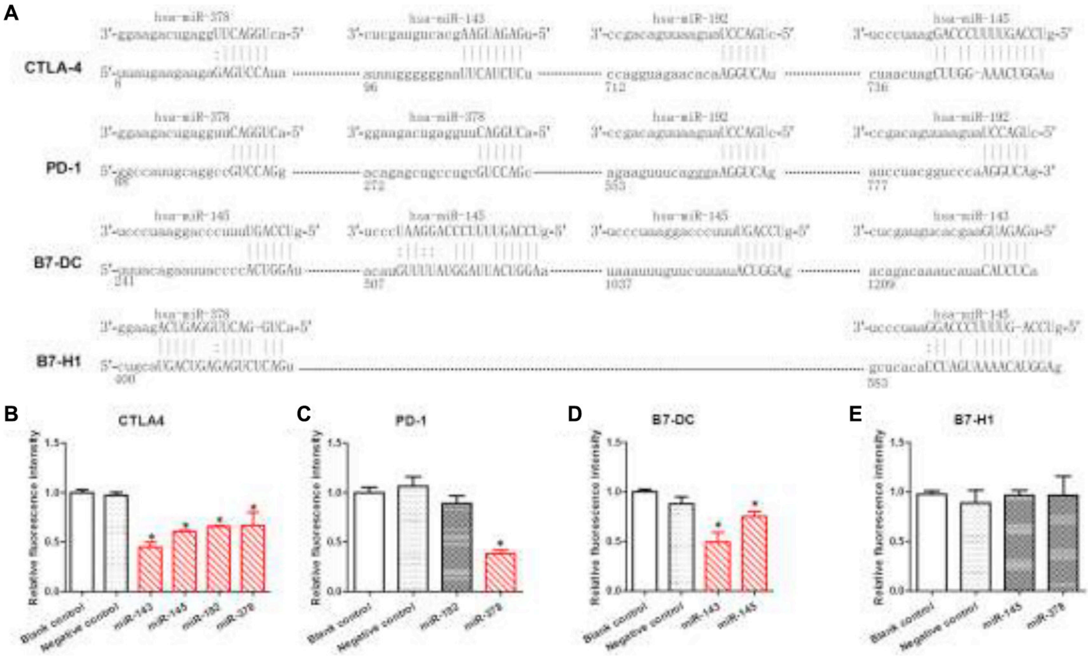

Supplementary Figure S3: Regulatory role of deregulated miRNAs in the expression of B7/CD28 molecules. (**A**) The predicted binding-sites of miRNAs in the 3′-UTRs of B7/CD28 genes. (**B**–**D**) The impact of miRNAs on the expression of CTLA4 (**B**), PD-1 (**C**), B7-DC (**D**), and B7-H1 (**E**).

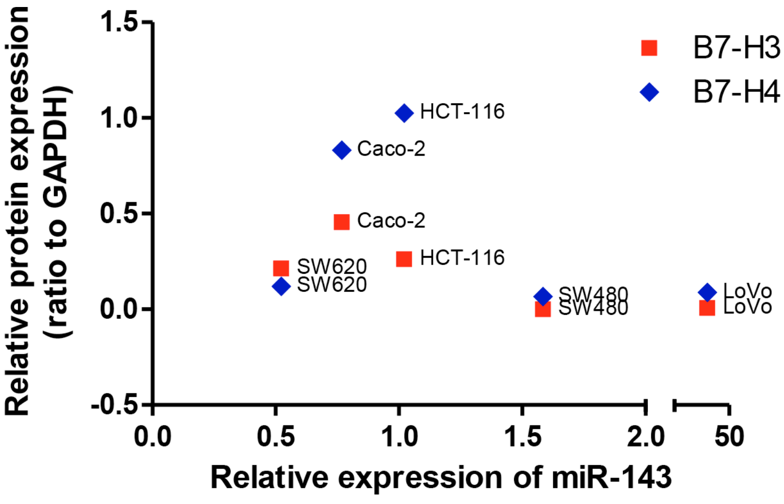

Supplementary Figure S4: The negative relationship between the expression of miR-143 and the expression of B7-H3 or B7-H4 protein in CRC cell lines.

Supplementary Table S1: The deregulated miRNAs and their verified targets.
See Supplementary_Table_S1

Supplementary Table S2: The deregulated miRNAs and their verified transcriptional factors and regulators. See Supplementary_Table_S2

Supplementary Table S3: The expression level of miRNAs and their verified targets

miRNA	N1	N2	N3	N4	N5	N6	r	Slope	Intercept
miR-155-5p	106.3318	85.7791	141.2022	12.8410	174.4965	208.7825			
miR-143-3p	1197.5060	1235.3920	755.3007	1960.9300	689.9388	527.9663	−0.9792	−7.3897	1959.5562
miR-28-5p	88.8509	100.7274	83.9470	111.4533	44.5270	44.9061	−0.9324	−0.3795	125.2087
miR-192-5p	1864.8570	2068.6560	1296.4480	2089.7417	1069.0510	899.3497	−0.9279	−7.0136	2400.6811
miR-145-5p	1208.4170	1165.7240	1061.0870	2409.4720	1003.6690	925.2208	−0.8682	−6.9418	2139.5239
miR-24-3p	1099.0060	1270.4320	1168.2920	1579.1590	1138.0490	1107.3420	−0.8512	−2.2460	1500.0924
miR-133a-3p	28.6480	26.5652	2.7789	90.4799	33.2486	16.9124	−0.7586	−0.3289	73.0939
miR-30a-3p	75.8615	73.7592	48.5377	165.2430	40.8582	81.7606	−0.7474	−0.4774	139.0374
miR-198	23.5632	22.2614	18.3171	39.5158	30.5901	16.7516	−0.6792	−0.0834	35.3098
miR-378a-3p	520.1780	549.1302	417.0721	716.9773	324.2951	591.1683	−0.6025	−1.1868	664.0825
miR-181a-5p	186.0108	168.3147	179.9921	25.6806	264.7472	237.0897	0.9226	1.1012	43.0961
miR-31-5p	8.7646	7.4585	12.5445	2.4705	11.7763	11.6827	0.9221	0.0505	2.9731
miR-93-5p	364.0481	294.9208	365.8606	217.2780	340.8476	390.1186	0.8860	0.8088	230.5206
miR-92a-3p	428.3228	407.2581	604.3593	391.2881	537.9766	541.7602	0.7872	0.9919	364.5722
miR-103a-3p	808.8664	771.4005	828.4414	812.6007	961.9224	953.9305	0.7842	0.9147	744.9867
miR-21-5p	62.0565	62.5222	105.9584	14.6244	91.1107	66.0038	0.7260	0.3268	27.3192
miR-18a-5p	90.1003	53.3095	89.3701	15.0135	45.5464	93.5444	0.6712	0.3071	27.1483
miR-20a-5p	370.8649	400.1153	451.0795	186.0181	409.6731	345.2568	0.6350	0.8474	257.4763

Supplementary Table S4: The KEGG enrichment analysis results

miRNAs	KEGG pathway	Counts	%	*P*-Value	Benjamini
mir-143, mir-145, mir-24, mir-23a, mir-200b, mir-148a, mir-132, mir-212, mir-183, mir-32, mir-125a, mir-149, mir-30a, mir-429, mir-193a, mir-181a, mir-630, mir-301a	TGF-beta signaling pathway	5	27.8	5.10E-05	2.80E-03
	Pancreatic cancer	4	22.2	7.00E-04	1.90E-02
	Chronic myeloid leukemia	4	22.2	7.90E-04	1.40E-02
	Pathways in cancer	6	33.3	9.10E-04	1.20E-02
	Colorectal cancer	4	22.2	1.10E-03	1.20E-02
	Cell cycle	4	22.2	3.50E-03	3.10E-02
	Adherens junction	3	16.7	1.60E-02	1.20E-01
	Cytokine-cytokine receptor interaction	4	22.2	2.60E-02	1.70E-01
	Wnt signaling pathway	3	16.7	5.50E-02	2.90E-01
	Jak-STAT signaling pathway	3	16.7	5.80E-02	2.80E-01
	Prion diseases	2	11.1	8.60E-02	3.60E-01
	Graft-versus-host disease	2	11.1	9.50E-02	3.70E-01

Supplementary Table S5: The synthetic small RNAs

RNA	Type	Forward primer (5′→3′)	Reverse primer (5′→3′)
miR-143-3p	mimics	UGAGAUGAAGCACUGUAGCUC	GCUACAGUGCUUCAUCUCAUU
	inhibitor	GAGCUACAGUGCUUCAUCUCA	
	agomir	UGAGAUGAAGCACUGUAGCUC	
miR-145-5p	mimics	GUCCAGUUUUCCCAGGAAUCCCU	GGAUUCCUGGGAAAACUGGACUU
miR-155-5p	mimics	UUAAUGCUAAUCGUGAUAGGGGU	CCCUAUCACGAUUAGCAUUAAUU
	inhibitor	ACCCCUAUCACGAUUAGCAUUAA	
miR-192-5p	mimics	CUGACCUAUGAAUUGACAGCC	CUGUCAAUUCAUAGGUCAGUU
miR-378a-3p	mimics	ACUGGACUUGGAGUCAGAAGGC	CUUCUGACUCCAAGUCCAGUUU
SMAD2	siRNA	GUCCCAUGAAAAGACUUAATT	UUAAGUCUUUUCAUGGGACTT
SMAD3	siRNA	CGUCAACACCAAGUGCAUCTT	GAUGCACUUGGUGUUGACGTT
SMAD4	siRNA	GAUGAAUUGGAUUCUUUAATT	UUAAAGAAUCCAAUUCAUCTT
CEBPB	siRNA	GAAGACCGUGGACAAGCACTT	GUGCUUGUCCACGGUCUUCTT
Negative control	mimics	UUCUCCGAACGUGUCACGUTT	ACGUGACACGUUCGGAGAATT
Negative control	inhibitor	UCUACUCUUUCUAGGAGGUUGUGA	
Negative control	siRNA	UUCUCCGAACGUGUCACGUTT	ACGUGACACGUUCGGAGAATT

Supplementary Table S6: The synthetic oligonucleotides used for construction of plasmids, sequencing, and qPCR. See Supplementary_Table_S6
