# Supplementary material for: TGF-β1 promotes colorectal cancer immune escape by elevating B7-H3 and B7-H4 via the miR-155/miR-143 axis
Source: Oncotarget. 2016 Sep 10;7(41):67196–211. doi: 10.18632/oncotarget.11950 (PMC5341868; doi:10.18632/oncotarget.11950)
Supplement: Supplementary file 2 [file oncotarget-07-67196-s002.docx]

**Supplemental Materials**

**Table S1**. The deregulated miRNAs and their verified targets

| **miRNA** | **Profile** | **Target Gene** |
| --- | --- | --- |
| hsa-let-7a-5p | Down | MPL, MYC, NKIRAS2, ITGB3, NF2, NRAS, KRAS, PRDM1, lin-41, TRIM71, FOXA1, NR1I2, VDR, RAVER2, HMGA2, HMGA1, AGO4, APP, E2F1, UHRF2, DICER1, HRAS, IGF2, LIN28A, CASP3, CASP8, CASP9, IL6, E2F2, CCND2, TMED7, CDKN1A, HAS2, EGFR |
| hsa-miR-1 | Down | CEBPA, MEF2A, GATA4, HCN4, HDAC4, FOXP1, HCN2, PTMA, MET, CAND1, HAND2, TMSB4X, KCNJ2, GJA1, FN1, XPO6, POGK, PGM2, TAGLN2, SERP1, LASP1, NETO2, ADAR, KCNE1, BDNF, G6PD, SOX6, NOTCH3, ATP6V1B2, LARP4, CNN3, PNP, SRXN1, KIF2A, HSPD1, HSPA4, PIM1, , PPP2R5A, PAX3, TWF1, TWF2, EDN1, PRKCE, FABP3, SNAI2, SOX9, SRF, MYOCD, SP1 |
| hsa-miR-100-5p | Down | IGF1R, PLK1, MMP13, FGFR3, ATM, BMPR2, FLT1, MTOR |
| hsa-miR-101-3p | Down | MYCN, ATXN1, EZH2, APP, FOS, STMN1, MCL1, FBN2, ARID1A, SUZ12, EED, PTGS2, ATM, ATP5B, DUSP1, SOX9, DNMT3A, FMR1, MEIS1 |
| hsa-miR-124-3p | Down | EFNB1, NR3C2, BACE1, ADIPOR2, MTPN, CEBPA, IL6R, ROCK2, RELA, CDK4, CDK6, AHR, SLC16A1, IQGAP1, SNAI2, LAMC1, CTDSP1, ITGB1, HMGA1, AR, RDH10, ELK3, CDK2, CCL2, PEA15, EZH2, NR3C1, VIM, SMYD3, E2F6, NFKBIZ, FXN, MECP2 |
| hsa-miR-1245a | Down | BRCA2 |
| hsa-miR-126-3p | Down | PITPNC1, IGFBP2, KRAS, SPRED1, PLK2, EGFL7, SLC45A3, CCNE2, RGS3, TOM1, HOXA9, MERTK, CRK, VEGFA, PIK3R2, VCAM1, IRS1, SOX2, TWF1, TWF2, PTPN7, DNMT1, SLC7A5, TEK, ADAM9, MMP7, CXCL12, PGR |
| hsa-miR-129-5p | Down | SOX4, NOTCH1, UBE2F, FMR1 |
| hsa-miR-132-3p | Down | SIRT1, CDKN1A, ARHGAP32, RB1, Mecp2, HBEGF, RASA1, CRK, TJAP1, TLN2, CCNA2, CCNB1 |
| hsa-miR-133a-3p | Down | HCN4, PNP, CACNA1C, HCN2, CASP9, KCNQ1, FSCN1, KCNH2, TAGLN2, LASP1, MSN, EGFR, VKORC1, PRDM16, EGFL7, VEGFA, PIK3R2, RGS3, COL1A1, GSTP1, SP1, BCL2L1, MCL1 |
| hsa-miR-137 | Down | PTGS2, E2F6, CDK6, NCOA2, KDM1A, CDC42, CTBP1, MITF, ESRRA, ZNF804A, YBX1, CSE1L, PXN |
| hsa-miR-138-5p | Down | PTK2, ARHGEF3, ROCK2, RHOC, H2AFX, SLC45A3, TERT, EID1, IGF1R, CCND3, SIRT1, HIF1A, CASP3, BLCAP, MXD1, RELN, EZH2, SOX4, CDH1, SNAI2, EED, SUZ12, ZEB2, VIM |
| hsa-miR-139-5p | Down | IGF1R, FOS |
| hsa-miR-140-5p | Down | HDAC4, VEGFA, PDGFRA, DNMT1, DNPEP, SOX2, OSTM1, FGF9, TGFBR1 |
| hsa-miR-143-3p | Down | MACC1, KRAS, MYO6, FHIT, DNMT3A, SERPINE1, FNDC3B, MAPK7, COL1A1, HRAS, FSCN1, HK2, AKT1, MDM2, BCL2, MMP13 |
| hsa-miR-145-5p | Down | BNIP3, SOX2, KLF4, MUC1, MYO6, CDKN1A, ITGB8, STAT1, YES1, CBFB, PPP3CA, CLINT1, IRS1, , IRS2, VEGFA, TMOD3, HOXA9, FSCN1, MYC, FLI1, IFNB1, TIRAP, POU5F1, IGF1R, PPM1D, MYRF, CPEB4, FZD7, ROBO2, SRGAP1, EIF4E, CDK4, SERPINE1, SWAP70, NEDD9, PAK4, DDX17, ERG, NRAS, ILK, CTGF, SOCS7, MDM2, ADAM17, CDH2, HDAC2, RTKN, F11R, ARL6IP5, AKR1B10, C11orf65, HLTF, GMFB, SERINC5, MEST, ALPPL2, NDRG2, DTD1, TPM3, MAP2K6, CEP19, TPRG1, GOLM1, CCDC43, MMP1, PTP4A2, TMEM9B, MMP12, MTMR14, ALDH3A1, NDUFA4, FAM3C, LYPLA2, FAM45A, PIGF, AP1G1, PHF17, NIPSNAP1, KREMEN1, MMP14, ABRACL, MIXL1, TSPAN6, PODXL, APH1A, ABHD17C, NANOG, MYO5A |
| hsa-miR-148a-3p | Down | DNMT1, HLA-G, TGIF2, DNMT3B, NR1I2, RPS6KA5, CCKBR, IRS1, ACVR1, BCL2, TMED7, CDC25B |
| hsa-miR-148b-3p | Down | HLA-G, CCKBR, ITGA5, ROCK1, PIK3CA, NRAS, CSF1 |
| hsa-miR-149-5p | Down | SP1, FOXM1 |
| hsa-miR-154-5p | Down | DICER1 |
| hsa-miR-15a-5p | Down | BMI1, WNT3A, MYB, CDC25A, CCND2, BCL2, CCND1, CCNE1, BACE1, DMTF1, BRCA1, AKT3, CADM1, TMEM184B, APP, UCP2, VEGFA, TSPYL2, CHUK, TP53, IFNG, PURA, RECK |
| hsa-miR-15b-5p | Down | CCNE1, RECK, BCL2, CCND1, VEGFA, EIF4A1, IFNG, PURA |
| hsa-miR-16-5p | Down | BMI1, HMGA1, ACVR2A, CDK6, CCND3, CCND1, CCNE1, PURA, PTGS2, ARL2, BCL2, CCNT2, ZYX, BRCA1, AKT3, WNT3A, VEGFA, CADM1, TPPP3, MYB, CAPRIN1, PPM1D, CHUK, TP53, NCOR2, PIM1, IFNG, UNG |
| hsa-miR-192-5p | Down | ERCC4, RB1, ACVR2B, BCL2, CDC7, CUL5, DLG5, DTL, ERCC3, HOXA10, HRH1, LMNB2, MAD2L1, MCM10, MIS12, KIF20B, PIM1, PRPF38A, RACGAP1, SEPT10, SMARCB1, TRAPPC2P1, CDKN1B, WNK1, ATP1B1 |
| hsa-miR-193a-5p | Down | TP73 |
| hsa-miR-195-5p | Down | WEE1, E2F3, CDK6, CCND1, CCND3, TBCCD1, CDK4, VEGFA, CCL4, BCL2, SLC2A3, CDC42, CAB39, CHUK, TAB3, MBD1, CCNE1, BCL2L2 |
| hsa-miR-198 | Down | CCNT1, NTRK3 |
| hsa-miR-199a-5p | Down | SMARCA2, EZH2, IKBKB, LIF, DDR1, EDN1, MAP3K11, SMAD4, SULT1E1, GPR78, ERBB2, UNG, CAV1, SIRT1 |
| hsa-miR-206 | Down | MET, NOTCH3, ESR1, , UTRN, FSTL1, TAC1, PAX3, Tppp, CCND2, GJA1 |
| hsa-miR-212-3p | Down | RB1, MECP2, TJP1, PEA15, PTCH1, KCNJ2, CCNA2, CCNB1 |
| hsa-miR-214-3p | Down | EZH2, XBP1, PTEN, MAP2K3, MAPK8, POU4F2, PLXNB1, SRGAP1, QKI, TWIST1, TP53, CTNNB1, ATF4, BCL2L2, SRGAP2, PSMD10, ASF1B |
| hsa-miR-215-5p | Down | ACVR2B, WNK1, CTNNBIP1 |
| hsa-miR-218-5p | Down | MBNL2, EFNA1, NUP93, MRPS27, EBP, LAMB3, RICTOR, LASP1, IKBKB, SP1, VOPP1, BIRC6, ACTN1, STAM2, CDKN1B, SOST, SFRP2, DKK2, TOB1, CDK6, BMI1 |
| hsa-miR-24-3p | Down | FEN1, CDK4, CCNA2, AURKB, MYC, E2F2, FAF1, NOTCH1, DHFR, MAPK14, HNF4A, TRIB3, ACVR1B, MLEC, CDKN2A, BRCA1, POLD1, CDKN1B, DND1, TGFB1, FURIN, ZNF217, ST7L, H2AFX, PAK4, CDK1, BCL2L11, DEDD, TMED7, STX16, DHFRP1, CHEK1, CORO1A, PCNA, PTPN9, PTPRF, SH3PXD2A, ARHGAP19 |
| hsa-miR-26b-5p | Down | CDK6, CCNE1, PTGS2, EPHA2, ABCA1, ARL4C |
| hsa-miR-28-5p | Down | E2F6, MAPK1, TEX261, OTUB1, N4BP1, CDKN1A, MPL |
| hsa-miR-29b-3p | Down | HDAC4, CTNNBIP1, COL5A3, COL1A1, SP1, CDK6, BACE1, PPP1R13B, SFPQ, DNAJB11, NASP, PTEN, DNMT3B, DNMT3A, MCL1, BCL2, DNMT1, S100B, VEGFA, ESR1, NCOA3, TET1, TCL1A, CDC42, Mmp15, MMP24, GRN, FGG, FGA, FGB, COL3A1, COL4A1, MMP2, ADAM12, NID1, BMP1, NKIRAS2, RAX, TBX21, IFNG, DUSP2, FOS, IMPDH1, MYCN |
| hsa-miR-30a-3p | Down | CDK6, SLC7A6, TMEM2, THBS1, CYR61, Fmr1, VEZT |
| hsa-miR-30b-5p | Down | SOCS1, BCL6, SMAD1, CCNE2, Snai1, CAT |
| hsa-miR-320a | Down | AQP4, AQP1, NPR1, TAC1, TFRC, MCL1, HSPB6, MAPK1 |
| hsa-miR-328-3p | Down | PTPRJ, BACE1, ABCG2, CD44, H2AFX |
| hsa-miR-338-3p | Down | UBE2Q1, NOVA1, MAP1A, DAB2IP, ZBTB18, PLA2G4B, SMO, CCND1, MMP2, MMP9 |
| hsa-miR-342-3p | Down | GEMIN4, BMP7, DNMT1 |
| hsa-miR-345-5p | Down | CDKN1A, ABCC1, NTRK3 |
| hsa-miR-34a-5p | Down | GRM7, JAG1, FOSL1, MYC, MYB, MET, CDK4, CCNE2, EPHA5, MYCN, SYT1, CCND3, CDC25A, CDK6, CCND1, ULBP2, IMPDH2, IMPA1, WNT1, AXIN2, E2F3, DLL1, VAMP2, NANOG, SIRT1, BCL2, NOTCH1, HNF4A, YY1, MAGEA12, MAGEA6, MAGEA3, MAGEA2, MAP2K1, PEA15, VEGFA, IFNB1, E2F1, NOTCH2, CD44, FOXP1, MAP3K9, CEBPB, SPI1, ZAP70, STX1A, SOX2, PDGFRA, AXL, CCL22, KLB, PPP1R10, BMP7, ACSL4, MTA2, LEF1, ACSL1, LDHA, CDKN2A, HDAC1, CDKN2C |
| hsa-miR-361-5p | Down | VEGFA |
| hsa-miR-375 | Down | TIMM8A, MTPN, PDK1, USP1, JAK2, ADIPOR2, C1QBP, PLAG1, MTDH, RASD1, YY1AP1, ELAVL4, MAP3K8, FZD8, YWHAZ, RHOA, KCNQ2 |
| hsa-miR-378a-3p | Down | SUFU, VEGFA, TUSC2, NPNT, GALNT7, MYC, TOB2, MSC, GRB2 |
| hsa-miR-409-3p | Down | PHF10, FGB, FGA, FGG, ANG, IFNG |
| hsa-miR-422a | Down | CYP8B1, CYP7A1 |
| hsa-miR-483-3p | Down | SMAD4, BBC3, Gmnn, Nhp2, Ube2c, PARD3 |
| hsa-miR-498 | Down | TERT, KRTAP5-9, RBFOX2 |
| hsa-miR-574-5p | Down | Dera, Nusap1, FOXN3 |
| hsa-miR-625-5p | Down | FHIT, NTRK3, ILK |
| hsa-miR-638 | Down | OSCP1 |
| hsa-miR-103a-3p | Up | CAV1, GPD1, CCNE1, CDK2, CREB1, DICER1, KLF4, DAPK1, PTEN, CYP2C8, TIMP3, ID2 |
| hsa-miR-106a-5p | Up | E2F1, FAS, CDKN1A, HIPK3, MYLIP, RB1, APP, RUNX1, ARID4B, VEGFA, IL10, CYP19A1, SIRPA |
| hsa-miR-10b-5p | Up | CDKN1A, TFAP2C, CDKN2A, BCL2L11, PIEZO1, HOXD10, TRA2B, SRSF1, KLF4, PPARA, NCOR2, NF1, TP53, NOTCH1, PAX6, MAPRE1, SDC1, NR4A3, NRP2 |
| hsa-miR-135a-5p | Up | JAK2, NR3C2, MYC |
| hsa-miR-135b-5p | Up | KLF4, MAFB, CASR |
| hsa-miR-155-5p | Up | UQCRFS1, MEIS1, TAB2, MECP2, SOCS1, MSH6, MSH2, MLH1, INPP5D, DET1, SMAD5, HIVEP2, ZNF652, ZIC3, BACH1, Arntl, Sla, Hif1a, Csf1r, Jarid2, Cebpb, Picalm, CSNK1A1, APC, TRIP13, TBCA, SMAD1, SDCBP, RHEB, POLE3, PKN2, PHC2, PDLIM5, NARS, MYO10, MSI2, DHX40, BRPF3, ARID2, ARFIP1, TM6SF1, MATR3, LDOC1, PHF17, RHOA, AGTR1, PKIA, TP53INP1, IKBKE, FGF7, KDM3A, NFATC2IP, SPI1, Cux1, Sfpi1, EDN1, FOXO3, TSHZ3, RUNX2, JUN, IFNGR1, KBTBD2, KRAS, ETS1, TLE4, FLI1, CYR61, ICAM1, SELE, SMAD2, MYB, SKI, GCSAM, IL13RA1, BCL6, MITF, MAP3K10, NOS3, ANAPC16, C17orf80, MYD88, GCFC2, EXOSC2, LNX2, ZNF248, CHD9, MEF2A, CAB39, CLUAP1, CARD11, PCDH9, ZNF561, CARHSP1, C16orf62, LIN7C, CBR4, GPM6B, LRIF1, TAF5L, HERC4, MORC3, MBNL3, UPF2, TSPAN14, INTS6, YWHAZ, PRKAR1A, SSX2IP, FAM199X, RAC1, PLS1, SAP30L, MRPS27, OLR1, SMAD4, CD68, CEP41, CIAPIN1, CCDC82, ACTR2, TRAK1, CYP2U1, SLC35F2, ZNF493, HAL, IL17RB, TBC1D14, ZNF254, GABARAPL1, IGJ, RAPGEF2, WBP1L, PBRM1, MRPL18, MAP3K14, ARMC2, LCORL, APAF1, MPP5, RAB11FIP2, NOVA1, RBAK, ARL15, MYO1D, LRRC59, SMAD3, TTF1, FAM91A1, CCDC41, KIAA0430, CDC40, DCUN1D2, KLHL5, AGO4, HBP1, WWC1, WEE1, GOLT1B, PALD1, IL8, ZNF83, PHF14, TBC1D8B, INPP5F, ARPC3, KRCC1, FAM177A1, UBTD2, SECISBP2, PAK2, SLC33A1, ZNF28, MCM8, SMARCA4, TCF12, TOMM20, UBQLN1, ZNF611, VPS18, WHSC1L1, MASTL, MYBL1, GATM, E2F2, FAM135A, C3orf18, ARL6IP5 |
| hsa-miR-17-5p | Up | ZNFX1, CCL1, GPR137B, NABP1, NPAT, YES1, JAK1, PTEN, CDKN1A, PTPRO, PKD2, BCL2L11, E2F1, MAP3K12, BCL2, MEF2D, RUNX1, APP, VEGFA, MAPK9, DNAJC27, FBXO31, TGFBR2, TNFSF12, BMPR2, CCND1, MYC, NCOA3, SMAD4, ICAM1, SELE, CCND2, E2F3, RB1, RBL1, RBL2, WEE1, RND3, TCF3, HSPB2, MMP2, HBP1, SIRPA |
| hsa-miR-181a-5p | Up | DUSP6, NLK, GATA6, CDX2, PRAP1, PLAG1, RALA, PTPN22, PTPN11, DUSP5, BCL2, PROX1, KAT2B, CDKN1B, ZNF763, DDIT4, ATM, HIPK2, BCL2L11, HRAS, SIRT1, FOS, MTMR3, KLF6, MCL1, XIAP, GPR78, NOTCH1 |
| hsa-miR-182-5p | Up | CDKN1A, FOXO3, MTSS1, FOXO1, RARG, MITF, ADCY6, CLOCK, TSC22D3, CREB1, CYLD, BCL2, CCND2, PDCD4, PFN1, SNAI2, RECK, SMAD4, FOXF2 |
| hsa-miR-183-5p | Up | EGR1, FOXO1, ITGB1, KIF2A, EZR, BTRC, PDCD4, AKAP12, IDH2 |
| hsa-miR-18a-5p | Up | ESR1, PTEN, CTGF, NCOA3, TNFSF11, NR3C1, TGFBR2, Prmt5, Myc, SMAD4, HSF2, ATM, Smad2, DICER1, SMAD3 |
| hsa-miR-196b-5p | Up | HOXB8, HOXC8, CD8A |
| hsa-miR-19a-3p | Up | HOXA5, MECP2, PTEN, ESR1, CCND1, ERBB4, NR4A2, ATXN1, KAT2B, SOCS1, PRMT5, BCL2L11, TGFBR2, BMPR2, SMAD4, CUL5, TLR2, SUZ12, RAB13, MSMO1, ABCA1, PSAP, DPYSL2, VPS4B, MYCN, RAB14 |
| hsa-miR-19b-3p | Up | BACE1, PTEN, ATXN1, HIPK3, ARID4B, MYLIP, ESR1, NCOA3, KAT2B, SOCS1, BCL2L11, BMPR2, CUL5, TLR2, CYP19A1, GCM1, MYCN |
| hsa-miR-200b-3p | Up | RND3, KDR, FLT1, VEGFA, PTPN12, ZEB2, BAP1, E2F3, ZEB1, RERE, ETS1, WASF3, ZFPM2, MATR3, CCNE2, BCL2, XIAP, SMAD2, CREB1, KLHL20, ELMO2, PTPRD, ERBB2IP, WDR37, TCF7L1, VAC14, HOXB5, RIN2, RASSF2, KLF11, SEPT7, SHC1 |
| hsa-miR-20a-5p | Up | HIF1A, CCND1, E2F1, BMPR2, CDKN1A, EGLN3, TGFBR2, MAP3K12, BCL2, MEF2D, PTEN, APP, RUNX1, NRAS, VEGFA, MYC, BNIP2, CCND2, E2F3, MAPK9, RB1, RBL1, RBL2, WEE1, PURA, ARHGAP12, TSG101, SIRPA |
| hsa-miR-210-3p | Up | HOXA9, TP53I11, PIM1, HOXA1, FGFRL1, RAD52, NPTX1, EFNA3, CASP8AP2, P4HB, PTPN1, BDNF, XIST, CPEB2, GPD1L, ISCU, NCAM1, E2F3, DDAH1, MRE11A, XPA, MNT, AIFM3, VMP1 |
| hsa-miR-211-5p | Up | TCF12, ELOVL6, RAB22A, POU3F2, CREB5, KCNMA1, CDH5, IL11 |
| hsa-miR-21-5p | Up | RASGRP1, CDC25A, BCL2, TM9SF3, RTN4, RPS7, PLOD3, NCAPG, DERL1, BASP1, JAG1, REST, SMARCA4, SPRY2, DUSP10, TIMP3, SOX5, MTAP, DOCK7, DOCK5, RECK, PIAS3, FMOD, TGFBR2, PTEN, E2F1, TGFBI, LRRFIP1, MARCKS, SP1, CCL20, TPM1, NFIB, APAF1, BTG2, PDCD4, RHOB, ANP32A, SERPINB5, BMPR2, TGIF1, NCOA3, ERBB2, PCBP1, JMY, TOPORS, HNRNPK, DAXX, TP53BP2, TP63, TGFBR3, PPIF, MSH2, MSH6, TIAM1, ISCU, MEF2C, EIF4A2, ANKRD46, EGFR, IL1B, ICAM1, PLAT, PTX3, TNFAIP3, CCR1, CDK2AP1, DOCK4, PPARA, FASLG, SOD3, TCF21, SMAD7, VEGFA, AKT2, STAT3, BCL6 |
| hsa-miR-221-3p | Up | CDKN1B, BMF, FOXO3, DICER1, KIT, CDKN1C, TMED7, HMGXB4, BBC3, ARIH2, USP18, BRAP, CREBZF, DKK2, MYBL1, TBK1, BNIP3L, DDIT4, TIMP3, TNFSF10, ICAM1, FOS, BNIP3, NAIP, ESR1, TICAM1, PTEN, SELE, TP53, CORO1A, TCEAL1, CERS2, FMR1, DVL2, MEOX2, ZEB2 |
| hsa-miR-223-3p | Up | MEF2C, STMN1, LMO2, EPB41L3, E2F1, RHOB, LIF, IGF1R, FBXW7, SLC2A4, NFIX, NFIA, Arid4b, Il6, Lpin2, CHUK, SP3, ARTN, FOXO1, HSP90B1, SCARB1, PARP1 |
| hsa-miR-22-3p | Up | PTMS, TCEAL1, ERBB3, ARPC5, BMP7, PPARA, ESR1, HDAC4, TFRC, MYCBP, ACVR1C, BDNF, HTR2C, MAOA, RGS2, Ogn, HMGB1, IRF5, RCOR1, SP1 |
| hsa-miR-23a-3p | Up | FOXO3, CXCL12, G6PC, HES1, PPARGC1A, POU4F2, Celf1, IL6R, FANCG, MYH1, MYH2, MYH4, PTEN, PTPN11, HMGN2 |
| hsa-miR-25-3p | Up | PRMT5, BCL2L11, CCL26, KLF4, CDKN1C, KAT2B, TP53, CDH1, MDM2, EZH2, SMAD7 |
| hsa-miR-29a-3p | Up | BCL7A, TNFAIP3, DICER1, CDK6, CDC42, RAN, BACE1, PXDN, PPP1R13B, MYCN, DNMT3A, DNMT3B, COL4A1, COL4A2, MCL1, BCL2, CD276, DKK1, NAV3, SFRP2, ITIH5, S100B, IMPDH1, GLUL, PPM1D, PIK3R1, KREMEN2, FGG, FGA, FGB, LPL, CPEB3, CPEB4, ADAMTS9, ITGA11, NASP, PTEN, ABL1, HBP1 |
| hsa-miR-301a-3p | Up | MEOX2, NKRF, SERPINE1, SMAD4, RUNX3 |
| hsa-miR-31-5p | Up | HIF1AN, RHOA, PPP2R2A, LATS2, SATB2, FOXP3, SELE, CASR, YY1, RET, NUMB, NFAT5, KLF13, JAZF1, HOXC13, ETS1, ITGA5, MPRIP, MMP16, RDX, CXCL12, ARPC5, FZD3, DMD, TIAM1, ICAM1, DKK1, DACT3, PRKCE, RASA1, STK40, MCM2, CDK1, CREG1, MLH1, MET, PRMT5, BCL2L11, KAT2B, TSC1, MDM2, SLC45A3 |
| hsa-miR-326 | Up | GLI1, SMO, NOTCH1, NOTCH2, MSH3, PKM, CD9 |
| hsa-miR-424-5p | Up | CCNF, CDC14A, CDC25A, CHEK1, KIF23, ATF6, WEE1, ANLN, PLAG1, CCNE1, CCND3, CDK6, CCND1, FGFR1, PIAS1, MAP2K1, ITPR1, NFIA, MYB, SIAH1, HIF1A, CUL2, SPI1 |
| hsa-miR-429 | Up | ZEB2, ZEB1, RERE, WASF3, ZFPM2, BCL2, XIAP, OSTF1, SOX2, KLHL20, PTPRD, ELMO2, ERBB2IP, BAP1, WDR37, VAC14, TCF7L1, HOXB5, RASSF2, RIN2, KLF11, SEPT7, SHC1, MYC |
| hsa-miR-494-3p | Up | PTEN, ARNTL, BCL2L11, PBX3, MEIS1 |
| hsa-miR-499a-5p | Up | SOX6, FOXO4, PDCD4 |
| hsa-miR-630 | Up | BCL2, BCL2L2, YAP1, SNAI2 |
| hsa-miR-92a-3p | Up | KLF2, ITGA5, ARID4B, HIPK3, MYLIP, TP63, KAT2B, ESR2, TGFBR2, BMPR2, CPEB2, OSBPL2, MAPRE1, RFFL, OSBPL8, PCGF5 |
| hsa-miR-93-5p | Up | TP53INP1, CDKN1A, E2F1, MAPK9, VEGFA, ITGB8, KAT2B, TUSC2, PTEN, PURA, LATS2 |
| hsa-miR-95-3p | Up | SNX1 |
