# Supplementary material for: TGF-β1 promotes colorectal cancer immune escape by elevating B7-H3 and B7-H4 via the miR-155/miR-143 axis
Source: Oncotarget. 2016 Sep 10;7(41):67196–211. doi: 10.18632/oncotarget.11950 (PMC5341868; doi:10.18632/oncotarget.11950)
Supplement: Supplementary file 3 [file oncotarget-07-67196-s003.docx]

**Table S2**. The deregulated miRNAs and their verified transcriptional factors and regulators

| **miRNA** | **Profile** | **Transcriptional factors and regulators** |
| --- | --- | --- |
| let-7a | Down | EIF2C2, FSH, MYC, TRIM32, E2F1, E2F3, LIN28, LIN28B, FLI1 |
| miR-1 | Down | CEBPA, MRF4, MYF5, MyoD, Myogenin, SLUG, YY1 |
| miR-101 | Down | KDM2A, EZH2 |
| miR-125a | Down | EGR1, TLR2 |
| miR-126 | Down | klf2a |
| miR-132 | Down | CREB1, EGR1, SP1, GNRH1 |
| miR-133a | Down | MRF4, MYF5, MyoD, Myogenin, calcineurin, TNFSF12 |
| miR-137 | Down | SOX2, Ezh2, MECP2 |
| miR-140 | Down | SOX9, sox9a |
| miR-143 | Down | FSH, IFNbeta, IFNgamma, Src, TP53, CEBPB, KLF2, TP73, SMAD3, SMAD4, JAG1, TGFB1 |
| miR-145 | Down | TP53, IFNbeta, IFNgamma, Src, KLF2, TP73, SMAD3, SMAD4, JAG1, BMP4 |
| miR-148a | Down | IL6, EGR1, MYB |
| miR-149 | Down | EGR1 |
| miR-15a | Down | c-myb, MYC, PRKCA, E2F1, E2F3, STAT5 |
| miR-15b | Down | FSH, E2F1, E2F3, STAT5 |
| miR-16 | Down | E2F1, E2F3, STAT5, NFKB1 |
| miR-192 | Down | TP53, TGFB1, HNF1A |
| miR-193a | Down | MAX, RXRA, TP63, TP73 |
| miR-195 | Down | MYC, EGR3, E2F1, E2F3, EGR3, STAT5 |
| miR-206 | Down | MRF4, MYF5, MyoD, Myogenin, TNFSF12, MyoD |
| miR-212 | Down | CREB1, EGR1, GNRH1 |
| miR-214 | Down | TWIST1, NFKB1 |
| miR-215 | Down | TP53 |
| miR-24 | Down | TGFB1, PDGF |
| miR-26b | Down | MYC |
| miR-29b | Down | NFKB1, YY1, CEBPA, MYC |
| miR-30a | Down | EGR1, ESR2 |
| miR-338 | Down | SOX10, SPI1 |
| miR-342 | Down | SPI1 |
| miR-34a | Down | MYC, TP53, NR1H4, CEBPA, NFKB1, SNAI1, ZEB1, E2F3 |
| miR-375 | Down | CTCF, ASH1L |
| miR-378 | Down | MYOD1 |
| miR-218 | Down | E6 |
| miR-106a | Up | E2F1, EGR1, MYC, MYCN, SP1, ERS1 |
| miR-10b | Up | PDLIM7, TWIST1, NFKB1, TWIST1 |
| miR-135b | Up | STAT3, FOXM1 |
| miR-155 | Up | BCR, FOXP3, NFKB1, SMAD4, TGFB1, cAMP, TP53, AKT1, SMG-1, MYB, IRF1, NFKB3, SPI1, AP-1 |
| miR-17 | Up | CCND1, E2F1, MYC, MYCN, NKX2-5, TLX1, TLX3, TNF, ERS1, STAT5, NFKB1, SPI1 |
| miR-181a | Up | TP63 |
| miR-182 | Up | IL2, DDX5 |
| miR-183 | Up | EGR1 |
| miR-18a | Up | E2F1, MYC, MYCN, NKX2-5, TLX1, TLX3, ERS1, STAT5, SPI1 |
| miR-196b | Up | ETS2 |
| miR-19a | Up | E2F1, MYC, MYCN, NKX2-5, PTEN, TLX1, TLX3, ERS1, STAT5, SPI1 |
| miR-19b | Up | E2F1, MYC, MYCN, NKX2-5, TLX1, TLX3, ERS1 |
| miR-200b | Up | SIP1, TGFB1, ZEB1, ZEB2, EGR1, BMP4, TWIST1, SLUG, SIX1, SMAD3, TP53, GATA3 |
| miR-20a | Up | CCND1, E2F1, MYC, MYCN, NKX2-5, TLX1, TLX3, ERS1, STAT5, SPI1 |
| miR-21 | Up | AP-1, BMP6, ERS1, NFIB, REST, Foxo3a, RAS/ERK, BMPR1a, BMPR1b, EGFR, PTEN, NFKB1, STAT3AP-1, NFKB1, DDX5, TCF4, ETV5, REL, RELA, STAT3 |
| miR-210 | Up | HIF1A, VHL |
| miR-211 | Up | MITF |
| miR-22 | Up | MYC, PTEN, AKT1, AKT2, AKT3 |
| miR-221 | Up | MYC, MYCN, PDGF, ERS1, SF2/ASF, p27, FOSL1 |
| miR-223 | Up | AML1, E2F1, CEBPA |
| miR-23a | Up | MYC, TGFB1, SPI1, RUNX2, EGR1 |
| miR-25 | Up | E2F1, PTEN |
| miR-29a | Up | HMGA1, MYC, NFKB1, YY1, IL-4, PDGF-B, TGFB1, CEBPA, AP-1 |
| miR-301a | Up | IL6 |
| miR-31 | Up | BMP2, TNF |
| miR-32 | Up | EGR1 |
| miR-424 | Up | HIF1A |
| miR-429 | Up | SIP1, TGFB1, ZEB1, ZEB2 |
| miR-630 | Up | TP63 |
| miR-92a | Up | E2F1, MYC, MYCN, NKX2-5, TLX1, TLX3, ERS1 |
| miR-93 | Up | E2F1, MYC |
