# Supplementary material for: TGF-β1 promotes colorectal cancer immune escape by elevating B7-H3 and B7-H4 via the miR-155/miR-143 axis
Source: Oncotarget. 2016 Sep 10;7(41):67196–211. doi: 10.18632/oncotarget.11950 (PMC5341868; doi:10.18632/oncotarget.11950)
Supplement: Supplementary file 4 [file oncotarget-07-67196-s004.docx]

**Table S6.** The synthetic oligonucleotides used for construction of plasmids, sequencing, and qPCR

| Gene | GenBank ID | Region | Experiment | Forward primer (5’→3’) | Reverse primer (5’→3’) |
| --- | --- | --- | --- | --- | --- |
| CD28 | NM_006139.3 | 3’-UTR | pGL3 construct | GGCTAGTCTAGAGCTCTGGATAGGAAATGACC | GGCTAGTCTAGACATCTTAGGTGGGCAGTGAC |
| CD80 | NM_005191.3 | 3’-UTR | pGL3 construct | GGCTAGTCTAGATAGCTCTGGTGACCTTGATC | GGCTAGTCTAGACTTCCCTTAGTATTGCTGAC |
| CD86 | NM_175862.4 | 3’-UTR | pGL3 construct | GGCTAGTCTAGAAAGGAGTTCTCATCCCTCTG | GGCTAGTCTAGAAAGCTTGTCTAGCATGGCAG |
| B7-H1 | NM_014143.3 | 3’-UTR | pGL3 construct | GGCTAGTCTAGACAGCATTGGAACTTCTGATC | GGCTAGTCTAGACTTGAATGGCTTGGAGGATG |
| B7-H2 | NM_015259.5 | 3’-UTR | pGL3 construct | GGCTAGTCTAGATTGGCTGTGATCCTGGAATG | GGCTAGTCTAGAAGTCAGGTTTCTGGAGATGG |
| B7-H3 | NM_001024736.1 | 3’-UTR | pGL3 construct | GGCTAGTCTAGATGTCTGTCTCATTGCACTGC | GGCTAGTCTAGAGACTATGCATCGTGTCTTTG |
|  |  | CDS | pcDNA3.1 | GGCTAGGGTACCATGCTGCGTCGGCGGGGCAGCCCTGGCATGGGTGTGCAT | CTAGCCTCTAGATTAATGGTGATGGTGATGATGTCAGGCTATTTCTTGTCCATCATCTTC |
|  |  | CDS | qPCR | GGTCAGCTTCTGTCCCTCTG | GGAGTCCTTGAGGGAGGAAC |
| B7-H4 | NM_024626.3 | 3’-UTR | pGL3 construct | GGCTAGTCTAGAACTCAGCTGGGGTGATTTCG | GGCTAGTCTAGATGTAATACAGTCACCGTGGC |
|  |  | CDS | pcDNA3.1 | GGCTAGGGTACCATGGCTTCCCTGGGGCAGATCCTCTTCTGGAGCATAATT | CTAGCCTCTAGATTAATGGTGATGGTGATGATGTTATTTTAGCATCAGGTAAGGGCTGAG |
|  |  | CDS | qPCR | CTTCTGCCTCTCAGCCCTTA | GAAATAGTTCTGTAGATCCCTGTTG |
| B7-DC | NM_025239.3 | 3’-UTR | pGL3 construct | GGCTAGTCTAGATGCTATCTGAACCTGTGGTC | GGCTAGTCTAGAATGAGGATGTGTCGAGGCAC |
| CTLA4 | NM_005214.4 | 3’-UTR | pGL3 construct | GGCTAGTCTAGATAGCTTTCTCCTCACAGCTG | GGCTAGTCTAGATTCTTTGGGCTGTGCCATTC |
| CTLA4 |  | 3’-UTR | pGL3 construct | GGCTAGTCTAGAGCTCAGGACACTAATACACC | GGCTAGTCTAGAACCTGCTGCCTTCTTCTGTC |
| ICOS | NM_012092.3 | 3’-UTR | pGL3 construct | GGCTAGTCTAGAACATACATCTTCTGCTGGTG | GGCTAGTCTAGATAGGCCCACCTATGTAAGTC |
| PD-1 | NM_005018.2 | 3’-UTR | pGL3 construct | GGCTAGTCTAGAACTCATGTCTCAATGCCCAC | GGCTAGTCTAGATGCAGTGTGTGGATGTGAGG |
| miR-143-3p | NR_029684.1 | miRNA | Reverse transcription | GTCGTATCCAGTGCAGGGTCCGAGGTATTCGCACTGGATACGACGAGCTA |  |
|  |  |  | qPCR | GCTGAGATGAAGCACTG | GTGCAGGGTCCGAGGT |
| miR-145-5p | NR_029686.1 |  | Reverse transcription | GTCGTATCCAGTGCAGGGTCCGAGGTATTCGCACTGGATACGACAGGGAT |  |
|  |  |  | qPCR | GTCCAGTTTTCCCAGGA | GTGCAGGGTCCGAGGT |
| miR-155-5p | [NR_030784.1](http://www.ncbi.nlm.nih.gov/nuccore/NR_030784.1) |  | Reverse transcription | GTCGTATCCAGTGCAGGGTCCGAGGTATTCGCACTGGATACGACACCCCT |  |
|  |  |  | qPCR | TTAATGCTAATCGTGATAGGG | GTGCAGGGTCCGAGGT |
| miR-192-5p | [NR_029578.1](http://www.ncbi.nlm.nih.gov/nuccore/NR_029578.1) |  | Reverse transcription | GTCGTATCCAGTGCAGGGTCCGAGGTATTCGCACTGGATACGACGGCTGT |  |
|  |  |  | qPCR | GCGCGCCTGACCTATGAATTG | GTGCAGGGTCCGAGGT |
| miR-378a-3p | [NR_029870.1](http://www.ncbi.nlm.nih.gov/nuccore/NR_029870.1) |  | Reverse transcription | GTCGTATCCAGTGCAGGGTCCGAGGTATTCGCACTGGATACGACGCCTTC |  |
|  |  |  | qPCR | ACTGGACTTGGAGTCA | GTGCAGGGTCCGAGGT |
| SMAD2 | NM_005901.5 | CDS | qPCR | GCCTTTACAGCTTCTCTGAACAA | ATGTGGCAATCCTTTTCGAT |
| SMAD3 | NM_005902.3 | CDS | qPCR | CCAGGGCTTTGAGGCTGTCTA | GCAAAGGCCCATTCAGGTG |
| SMAD4 | NM_005359.5 | CDS | qPCR | TGGCCCAGGATCAGTAGGT | CATCAACACCAATTCCAGCA |
| CEBPB | NM_005194.3 | CDS | qPCR | CTCGCAGGTCAAGAGCAAG | GACAGCTGCTCCACCTTCTT |
| TP53 | NM_000546.5 |  |  | TAACAGTTCCTGCATGGGCGGC | AGGACAGGCACAAACACGCACC |
| GAPDH | NM_002046.5 | mRNA | qPCR | TGCACCACCAACTGCTTAGC | GGCATGGACTGTGGTCATGAG |
| U6 | NR_004394.1 | ncRNA | qPCR | CTCGCTTCGGCAGCACA | AACGCTTCACGAATTTGCGT |
